# Novel Introner-Like Elements in fungi Are Involved in Parallel Gains of Spliceosomal Introns

**DOI:** 10.1371/journal.pone.0129302

**Published:** 2015-06-05

**Authors:** Jérôme Collemare, Henriek G. Beenen, Pedro W. Crous, Pierre J. G. M. de Wit, Ate van der Burgt

**Affiliations:** 1 Laboratory of Phytopathology, Wageningen University, Wageningen, The Netherlands; 2 Present address: UMR1345 IRHS-INRA, Beaucouzé, France; 3 Present address: Dyadic, Wageningen, The Netherlands; 4 Evolutionary Phytopathology, CBS-KNAW Fungal Biodiversity Centre, Utrecht, The Netherlands; Illinois Institute of Technology, UNITED STATES

## Abstract

Spliceosomal introns are key components of the eukaryotic gene structure. Although they contributed to the emergence of eukaryotes, their origin remains elusive. In fungi, they might originate from the multiplication of invasive introns named Introner-Like Elements (ILEs). However, so far ILEs have been observed in six fungal species only, including *Fulvia fulva* and *Dothistroma septosporum* (*Dothideomycetes*), arguing against ILE insertion as a general mechanism for intron gain. Here, we identified novel ILEs in eight additional fungal species that are phylogenetically related to *F*. *fulva* and *D*. *septosporum* using PCR amplification with primers derived from previously identified ILEs. The ILE content appeared unique to each species, suggesting independent multiplication events. Interestingly, we identified four genes each containing two gained ILEs. By analysing intron positions in orthologues of these four genes in Ascomycota, we found that three ILEs had inserted within a 15 bp window that contains regular spliceosomal introns in other fungal species. These three positions are not the result of intron sliding because ILEs are newly gained introns. Furthermore, the alternative hypothesis of an inferred ancestral gain followed by independent losses contradicts the observed degeneration of ILEs. These observations clearly indicate three parallel intron gains in four genes that were randomly identified. Our findings suggest that parallel intron gain is a phenomenon that has been highly underestimated in ILE-containing fungi, and likely in the whole fungal kingdom.

## Introduction

Spliceosomal introns are hallmarks of the eukaryotic gene structure that are involved in regulation of gene expression and diversification of the protein repertoire [1–3]. Despite their predicted key role in emergence and evolution of eukaryotes, their origin is still enigmatic. Studies that attempted to trace the evolutionary history of introns since the last eukaryotic common ancestor (LECA) provided only a few hints. Indeed, modeling of intron dynamics, as well as intron gain and loss analyses across eukaryotes, indicate that intron losses prevail, suggesting that LECA was intron-rich [4–6]. However, these studies also suggest bursts of intron gains in certain lineages like in the ancestor of metazoans [6]. This indicates that intron gain might have prevailed in some lineages at particular times during evolution. For example, recent population genomics studies showed large-scale intron gains in the crustacean *Daphnia pulex* [7,8] and in the fungus *Zymoseptoria tritici* [9]. However, these events occur infrequently and so far reports on intron gains are scarce compared to those on intron losses. In this regard, complex presence-absence patterns of introns in orthologous genes are usually considered to be an indication of evolutionary conservation [10,11]. Accordingly, only recurrent losses explained complex presence-absence patterns in Angiosperms, and no recurrent gains could be inferred [12]. Therefore, independent gains of introns at the same position, also known as parallel gains, are expected to occur at an extremely low rate.

The discussion on the origin of spliceosomal introns has recently received new perspectives with the discovery of Introner Elements (IEs) in the alga *Micromonas pusilla* [13,14] and of Introner-Like Elements (ILEs) in six fungal species belonging to the *Dothideomycetes* [15]. Both IEs and ILEs are spliceosomal introns that have invaded algal and fungal genomes, where they are present in hundreds of near-identical copies in unrelated genes. Consistent with this observation, ILEs were shown to represent up to 90% of the most recent single intron gains in the fungus *Zymoseptoria tritici* [16], whereas other types of intron duplication, including tandem duplication within the same gene, contributed to less than 1% of single intron gains [16]. Similarly, more than 500 novel IE insertions were observed in marine metagenomes [14].

Identification of ILEs can be challenging because they rapidly become indistinguishable from regular spliceosomal introns (RSIs), which supports the hypothesis that ILEs are their predecessors [15]. ILE multiplication is likely still ongoing in *Fulvia fulva* (formerly *Cladosporium fulvum*) and *Z*. *tritici* because their genomes contain ILEs that are 99% and 98% identical, respectively. In contrast, ILEs in the genome of *Dothistroma septosporum* might have lost their ability to multiply because their pairwise identity is much lower [15]. In each fungus, ILEs could be grouped in up to eight different families based on sequence identity [15]. In a given species, some ILE families still comprise active elements with high pairwise identity while others do not. For example in *Z*. *tritici*, ILEs from the *mg01* and *mg02* families are likely active, while the *mg05* and *mg06* families seem to have lost their ability to multiply [15]. Thus, some ILEs appear still mobile, resulting in contemporary intron gains in several fungal species.

Here, we provide evidence for the presence of novel ILEs in eight non-sequenced fungal species using PCR amplification with primers derived from previously identified ILEs. Strikingly, we found parallel gains of ILEs at positions occupied by RSIs in distant fungal species. Our study shows that ILEs occur in many fungal species and can be responsible for parallel intron gains.

## Materials and Methods

### Phylogeny of fungal species

Internal transcribed spacer (ITS) and partial large-subunit (LSU) ribosomal DNA sequences of *F*. *fulva*, *D*. *septosporum*, *Pseudocercospora fijiensis*, *Z*. *tritici* and *Aspergillus niger* were retrieved from the Joint Genome Institute MycoCosm portal [17] (http://genome.jgi.doe.gov; August 29, 2013) with BLASTN using sequences from S1 Table as query.

All DNA sequences (S1 File) were aligned using MUSCLE [18] and poorly aligned regions were removed with Gblocks [19], allowing smaller final blocks, gap positions within the final blocks and less strict flanking positions. The edited alignment was used to construct a maximum-likelihood phylogenetic tree using all sites in MEGA5 [20]. The Hasegawa-Kishino-Yano substitution model (uniform rate) was used and a hundred bootstrap replications were performed.

### Genomic DNA isolation and PCR

Fungal strains obtained from CBS-KNAW were grown in 50 mL PDB (BD) for 7 to 9 days at 22°C, shaking at 150 rpm. Mycelium was retrieved by filtering through miracloth, rinsed with water and snap frozen in liquid nitrogen. Mycelium was then ground with a tissue lyser (Retsch mix miller). About 100 mg of powder were incubated in 750 μL of CTAB buffer (2% CTAB, 100 mM Tris pH 8, 20 mM EDTA and 1.4 M NaCl) for 1h at 57°C. Then 750 μL of phenol/chloroform were added, and tubes were centrifuged at 10,000 x g for 10 min. The aqueous phase was transferred to a new tube and a second extraction was performed using 500 μL of phenol/chloroform. Tubes were centrifuged at 10,000 x g for 10 min and the aqueous phase was transferred into a new tube. Genomic DNA was precipitated with 750 μL of isopropanol, and tubes were centrifuged at 4,500 x g for 10 min. Supernatant was discarded and the pellet was washed with 70% ethanol. After centrifugation at 4,500 x g for 5 min, pellet was air-dried and dissolved in 100 μL of TE overnight at 4°C. RNA was removed by adding 1 μL of RNase A (10 mg/mL), incubating at 37°C for 2h. Genomic DNA was purified adding 200 μL of phenol/chloroform. Tubes were centrifuged for 10 min at 10,000 x g and the aqueous phase was transferred into a new tube. DNA was precipitated by adding 200 μL of isopropanol and centrifugation at 4,500 x g for 10 min. Pellet was washed with 70% ethanol, centrifuged at 4,500 x g for 5 min and air-dried. The pellet was dissolved in 100 μL of water. Concentration was evaluated using a Nanodrop 1000 spectrophotometer (Thermo Scientific).

To design PCR primers for each family, ILEs exhibiting more than 80% pairwise identity (according to van der Burgt *et al*., 2012) were aligned with MUSCLE [18]. Conserved nucleotide stretches that might serve as primers were identified. Primers could be designed to specifically amplify ILEs from *cf01* and *cf02* families (S2 Table). For ILE families that are shared by *F*. *fulva* and *D*. *septosporum*, degenerated primers had to be designed, using sequence stretches with at most four variable nucleotides (S2 Table).

About 50 ng of genomic DNA were used as template for PCR using GoTaq Flexi (Promega), 1 mM MgCl_2_ and 5 pmol of each primer (S2 Table). After 2 min at 95°C, 35 cycles of the following program were used: 1 min at 95°C, 1 min at 55°C and 1 min at 72°C, followed by a final step of 5 min at 72°C.

### PCR fragment purification and sequencing

PCR products were either run on 2% agarose gels or 20% acrylamide gels (run for 7–8h at 50 V). Acrylamide gels were stained with 5,000 times diluted GelRed for 30 min shaking. Gels were washed with water for 15 min shaking prior to imaging.

PCR fragments were purified from agarose gels using the Wizard Gel and PCR clean-up system kit (Promega) according to the manufacturer’s recommendations. Fragments were cloned into PGEM-T vector (Promega) and transformed into DH5α *Escherichia coli* cells [21]. Plasmids were recovered from eight white colonies and insert size was checked by restriction digestion. Plasmids with inserts of different sizes were sent for sequencing (Macrogen).

PCR fragments were also recovered from acrylamide gels. Cut gel slices were crushed in tubes and two volumes of elution buffer were added (3.85 g ammonium acetate, 0.215 g magnesium acetate, 200 μL 0.5 M EDTA, 1 mL 10% SDS in 100 mL water). Tubes were incubated at 37°C for 4h on a rotating wheel and then centrifuged at 10,000 x g for 1 min at 4°C. Supernatant was transferred into a new tube and 0.5 volume of elution buffer was added to the pellet. After brief vortex, tubes were centrifuged at 10,000 x g for 1 min at 4°C. Both supernatants were combined and two volumes of cold 100% ethanol were added. After incubation on ice for 30 min, DNA was pelleted by centrifugation at 10,000 x g for 10 min at 4°C. Supernatant was discarded and pellet dissolved in 200 μL of TE. DNA was purified by adding 25 μL of 3 M sodium acetate pH 5.2 and two volumes of cold 100% ethanol. After incubation on ice for 30 min, DNA was recovered by centrifugation at 10,000 x g for 10 min at 4°C. Supernatant was discarded and pellet washed with 70% ethanol and centrifuged at 10,000 x g for 1 min at 4°C. The pellet was air-dried and dissolved in 10 μL of TE. 1 μL of this purified DNA fragment was used for a PCR reaction as described above, which was directly sent for sequencing (CBS; fragments and PCR primers mixed together with a BigDye Terminator Cycle Sequencing Kit v. 3.1 (Applied Biosystems) and analysed on an ABI Prism 3100 DNA Sequencer (Perkin-Elmer) [22]).

Sequences obtained from the PCR fragments were trimmed (S2 File) and aligned to the consensus sequences of ILE families using Muscle [18]. Obtained alignments were edited in GeneDoc [23].

### Identification of orthologues and intron landscape analysis

When two different ILEs were amplified in a PCR fragment, the exonic sequence in between was used as a query for a BLASTN search (word size 4, no filtering for low complexity regions and performing gapped alignment) in the masked assemblies of *F*. *fulva* and *D*. *septosporum* (http://genome.jgi.doe.gov) [24] and in the nr database at NCBI (www.ncbi.nlm.nih.gov). The predicted protein sequence of Fulfu186212, Fulfu193200, Dotse59237 and Zymtr43851, in which new ILE insertions were detected, were used to search for homologues in all Dikarya fungal genomes available at the JGI MycoCosm portal using BLASTP (October 4, 2013) [17]. All hits were aligned using Muscle [18]. The obtained alignments were used to manually remove predicted proteins that contained large deletions or insertions. This step has been iteratively performed until alignments of predicted proteins of similar length were obtained. These alignments were then used to construct minimum evolution phylogenetic trees in MEGA5 [20], using default parameters with the Jones-Taylor-Thornton (JTT) amino acid substitution model, 100 bootstraps replications and gap site complete deletion. Phylogenetic trees were used to discriminate between orthologues and paralogues. Distant homologues were removed and new alignments and phylogenetic trees were constructed in order to confidently identify orthologues. Putative orthologues from Basidiomycota were used as outgroup. The final alignments and phylogenetic trees were performed as described above, with the exception that a maximum likelihood tree was built, using all sites.

For each selected protein, the genomic DNA sequence was retrieved at JGI, from the predicted start to stop codons. DNA sequences were aligned using MUSCLE [18] and manually edited in GeneDoc [23] in order to correct misaligned exon-intron-exon boundaries. This step is particularly needed when two introns are separated by only a few nucleotides in different fungal species. Intron positions were determined using DNA alignments and mapped onto protein alignments. Conserved domains were sought in the NCBI Conserved Domain Database [25] using the predicted protein sequence of Fulfu186212, Fulfu193200, Dotse59237 and Zymtr43851.

## Results and Discussion

### Specific amplification of Introner-Like Elements in non-sequenced fungal species

**Fig 1:**
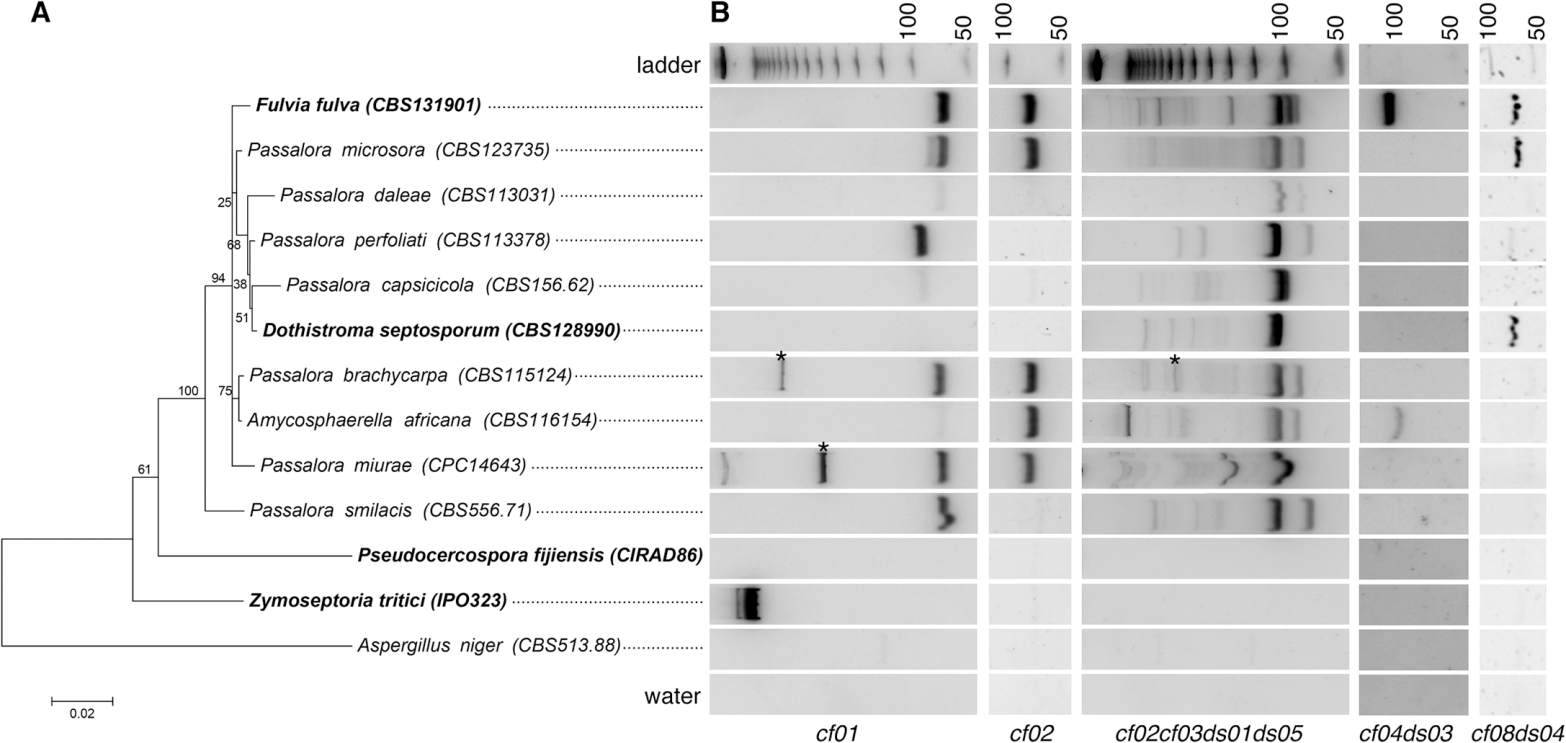
Detection of Introner-Like Elements (ILEs) in closely related dothideomycetous fungal species. (A) Maximum-likelihood phylogenetic tree using ITS and LSU sequences. Fungal species in which ILEs have been previously identified are highlighted in bold. *Aspergillus niger* belongs to the *Eurotiomycetes* and serves to root the tree. Accession numbers of fungal species from the CBS-KNAW collection are indicated in between brackets. The scale bar indicates the number of substitutions per site. (B) PCR with primers specific to single (*cf01* and *cf02*) or shared (*cf02cf03ds01ds05*, *cf04ds03* and *cf08ds04*) ILE families between *F*. *fulva* and *Dothistroma septosporum* was performed using genomic DNA. PCR products were run on 20% acrylamide gels. The first row shows a 50 bp-step DNA ladder and the last row shows the water control. Asterisks indicate fragments that correspond to two different ILEs as revealed by sequencing.

**Fig 2:**
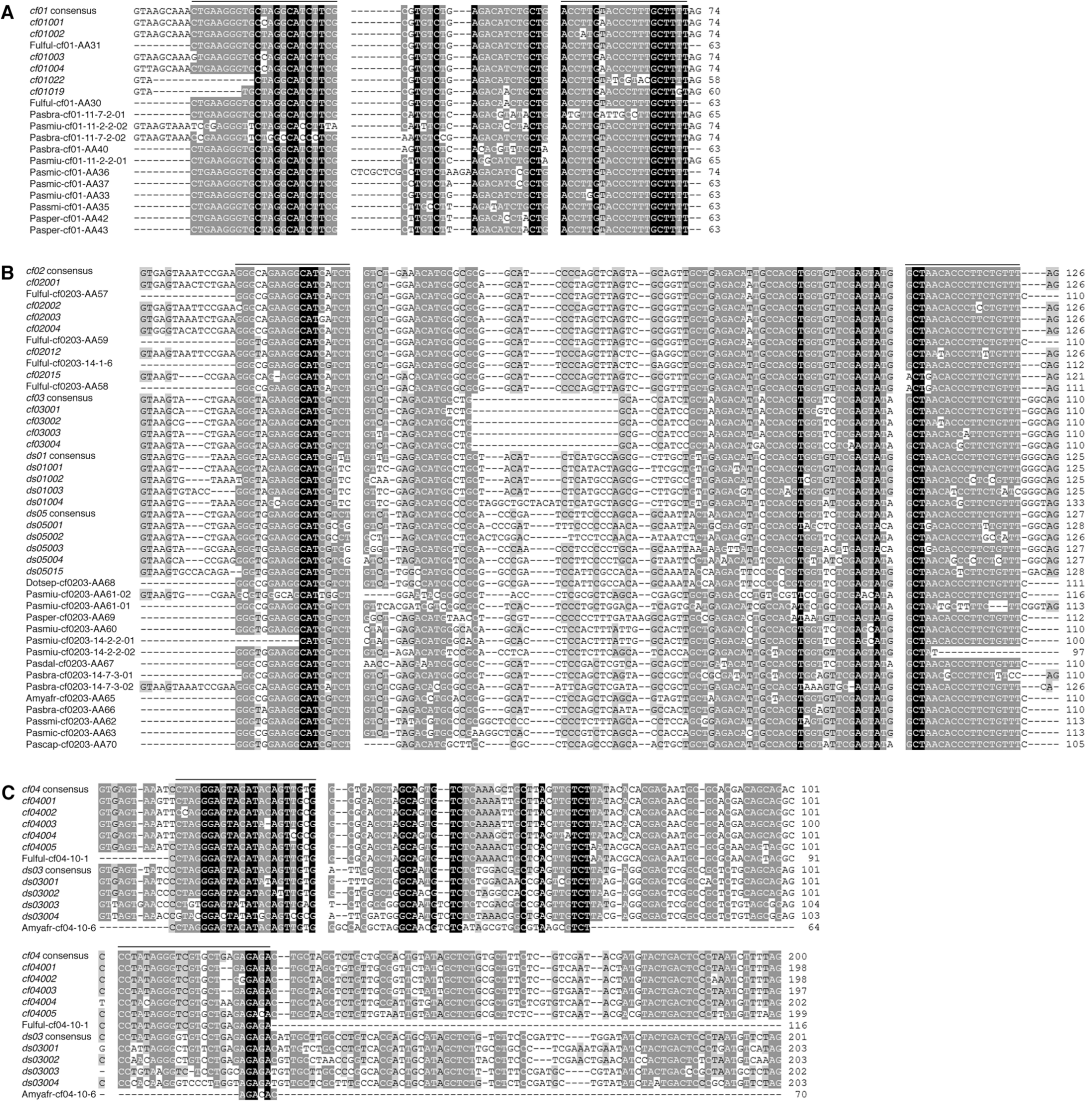
Alignments of newly discovered Introner-Like Elements (ILEs). DNA sequences obtained from the PCR fragments were aligned, using as references the consensus sequence and the four most conserved ILE sequences of the corresponding families in *F*. *fulva* and *Dothistroma septosporum*. The complete sequences of known ILEs amplified by PCR in this study are also included. Alignments are shown for ILEs related to (A) *cf01*, (B) *cf02cf03ds01ds05* and (C) *cf08ds04* families. Bars above the alignments indicate the oligonucleotide sequences. *Fulful*: *Fulvia fulva*; *Dotsep*: *Dothistroma septosporum*; *Amyafr*: *Amycosphaerella africana*; *Pasbra*: *Passalora brachycarpa*; *Pascap*: *Passalora capsicicola*; *Pasdal*: *Passalora daleae*; *Pasmic*: *Passalora microsora*; *Pasmiu*: *Passalora miurae*; *Pasper*: *Passalora perfoliati*; *Passmi*: *Passalora smilacis*.

*F*. *fulva* and *D*. *septosporum* are closely related fungal species with different ILE activities, as suggested by the higher pairwise identity of certain ILE families in *F*. *fulva* [15]. However, both species also share some ILE families [15]. We hypothesized that ILEs with significant sequence similarity to elements from described families might be present in other related fungal species. Thus, we assessed by PCR on genomic DNA the presence of ILEs in eight non-sequenced fungal species that are phylogenetically related to *F*. *fulva* and *D*. *septosporum* (Fig 1A). Genomic DNA of the latter two species was used as positive controls and DNA of the more distantly related species *Pseudocercospora fijiensis* (formerly *Mycosphaerella fijiensis*), *Z*. *tritici* and *Aspergillus niger* as negative controls. Specific oligonucleotides were designed to amplify ILEs related to *F*. *fulva cf01* and *cf02* families; degenerated oligonucleotides were designed to amplify ILEs related to families shared by *F*. *fulva* and *D*. *septosporum* (*cf02cf03ds01ds05*, *cf04ds03* and *cf08ds04*) (S2 Table). As expected, all oligonucleotide pairs amplified fragments in *F*. *fulva*, but not in the negative controls, proving their specificity (Fig 1B). The large fragment amplified in *Z*. *tritici* with the *cf01* oligonucleotide pair corresponds to the 5’UTR and start of the gene containing the *mg01020* ILE as revealed by sequencing (S2 File). The *cf01* and *cf02* pairs did not amplify fragments in *D*. *septosporum*, again proving their specificity. Degenerated oligonucleotide pairs amplified fragments in both *F*. *fulva* and *D*. *septosporum*, except for the *cf04ds03* pair that is more specific to *F*. *fulva* ILEs (Fig 1B). PCR results obtained for other fungal species revealed diverse amplification profiles, suggesting the presence of ILEs in these non-sequenced genomes (Fig 1B). Sequencing of nearly all PCR fragments shown in Fig 1B confirmed that they are true ILEs (Fig 2 and S2 File). It is noteworthy that the acrylamide gel resolution did not allow the separation of ILEs that differ in only a few nucleotides in length, as shown by the cloning of different *F*. *fulva* ILEs from the same PCR fragment (Fig 2A). In addition to fragments of the expected sizes corresponding to the different ILE families [15], larger fragments were also amplified with the *cf01* and *cf02cf03ds01ds05* oligonucleotide pairs. These likely correspond to related ILEs of different size or to different ILEs inserted in the same locus. Remarkably, the amplification profile of each fungal species is unique (Fig 1B) and all amplified ILEs differed in sequence (Fig 2). Moreover, for a given family, signal intensity of the PCR fragments varied, suggesting either degenerated ILEs or fewer ILEs in species like *Passalora daleae*, *Passalora capsicicola*, *Amycosphaerella africana* and *Passalora smilacis* (Fig 1B). Altogether, these results show that ILEs are highly dynamic and that many likely represent unique intron gains.

### Identification of genes with recent multiple ILE insertions

**Fig 3:**
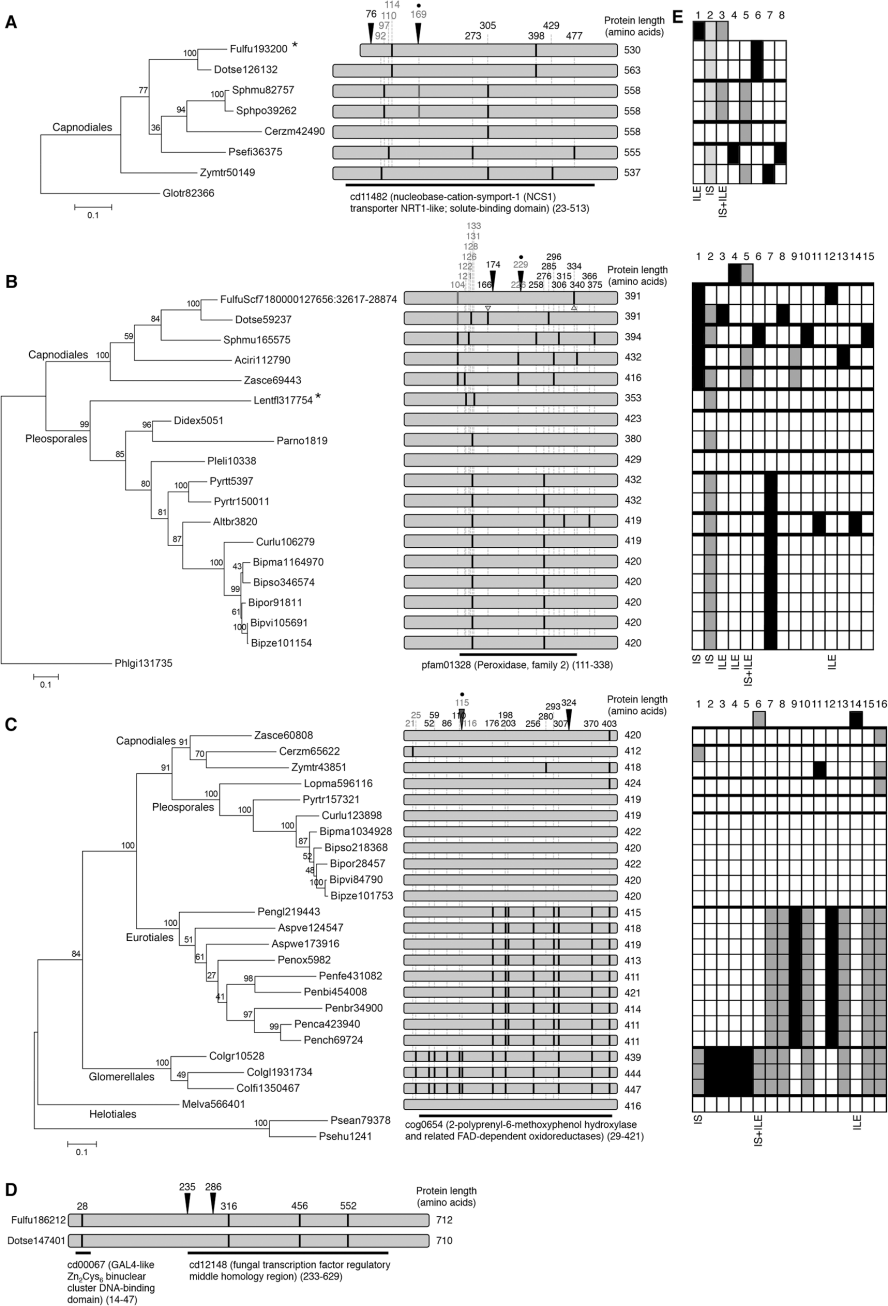
Analysis of intron positions in genes with recent insertions of Introner-Like Elements (ILE). ILEs from *Passalora brachycarpa* inserted in genes encoding (A) a transporter and (B) a peroxidase. ILEs from *Passalora miurae* inserted in genes encoding (C) a hydroxylase/oxidoreductase and (D) a fungal transcription factor. For each gene, a maximum likelihood phylogenetic tree was constructed with the predicted protein sequence of orthologues. The trees were rooted with the closest homologue found in Basidiomycota. Bootstrap values of 100 repeats are shown. Scale bar represents the number of substitutions per site. The numbers correspond to the protein ID from the Joint Genome Institute mycocosm portal, except for one gene that was not predicted in the *Fulvia fulva* genome and for which genomic coordinates are given (B). Orders in fungal classification are mentioned in the trees. On the right, diagrams depict aligned protein sequences and intron positions are indicated as black bars. Their positions in the protein alignment that served to build the phylogenetic trees are indicated above. Positions that are shown in grey highlight putative intron sliding. The black arrows indicate the positions where ILEs inserted in the genes of *P*. *miurae* or *P*. *brachycarpa*. The open triangles indicate previously identified ILEs. Dots indicate positions where parallel intron gains have occurred. The black bar below each protein representation indicates conserved domains (positions in the protein alignments are indicated between brackets). Asterisks behind species names indicate genes that are likely pseudogenes because of an in frame stop codon. (E) Schematic overview of intron positions (numbered on top) in three genes. The first row shows intron positions in genes of *P*. *miurae* or *P*. *brachycarpa*. Thick lines indicate monophyletic clades according to the phylogenetic trees. Black, dark grey and light grey squares indicate single presence of an intron position in a monophyletic clade, presence-absence polymorphism and single absence of an intron position in a monophyletic clade, respectively. White squares indicate absence of intron. The presence of ILE and occurrence of putative intron splicing (IS) are indicated below each scheme. *Aciri*: *Acidomyces richmondensis*; *Altbr*: *Alternaria brassicicola*; *Cerzm*: *Cercospora zeae-maydis*; *Fulfu*: *Fulvia fulva*; *Bipze*: *Bipolaris zeicola*; *Bipma*: *Bipolaris maydis*; *Curlu*: *Curvularia lunata*; *Bipor*: *Bipolaris oryzae*; *Bipso*: *Bipolaris sorokiniana*; *Bipvi*: *Bipolaris victoriae*; *Didex*: *Didymella exigua*; *Dotse*: *Dothistroma septosporum*; *Lentfl*: *Lentithecium fluviatile*; *Pleli*: *Plenodomus lingam*; *Lopma*: *Lophiostoma macrostomum*; *Psefi*: *Pseudocercospora fijiensis*; *Pyrtr*: *Pyrenophora tritici-repentis*; *Pyrtt*: *Pyrenophora teres f*. *teres*; *Sphmu*: *Sphaerulina musiva*; *Sphpo*: *Sphaerulina populicola*; *Parno*: *Parastagonospora nodorum*; *Zasce*: *Zasmidium cellare*; *Zymtr*: *Zymoseptoria tritici*; *Aspve*: *Aspergillus versicolor*; *Aspwe*: *Aspergillus wentii*; *Penbi*: *Penicillium bilaiae*; *Penbr*: *Penicillium brevicompactum*; *Penca*: *Penicillium canescens*; *Pench*: *Penicillium chrysogenum*; *Penfe*: *Penicillium fellutanum*; *Pengl*: *Penicillium glabrum*; *Penox*: *Penicillium oxalicum*; *Colgr*: *Colletotrichum graminicola*; *Colfi*: *Colletotrichum fiorinae*; *Colgl*: *Colletotrichum gloeosporioides*; *Melva*: *Meliniomyces variabilis*; *Glotr*: *Gloeophyllum trabeum*; *Phlgi*: *Phlebiopsis gigantean*; *Psean*: *Pseudozyma antarctica*; *Psehu*: *Pseudozyma hubeiensis*.

Sequencing of four larger *cf01* and *cf02cf03ds01ds05* fragments from *Passalora brachycarpa* and *Passalora miurae* revealed in each case the presence of two ILEs located next to each other (between 145 and 626 bp apart; Fig 1B, Fig 3A–3D and S2 File). The exonic sequences between the two ILEs correspond to four different genes that putatively encode a nucleoside transporter, a peroxidase, a hydroxylase/oxidoreductase and a fungal transcription factor, respectively. All four genes belong to multigene families because many paralogues could be identified, even in distant species belonging to the Basidiomycota. However, only a few orthologues could be assigned confidently, mainly within the *Dothideomycetes* (Fig 3A–3D and S3 File). An intron landscape was determined for each of the four genes by mapping all intron positions identified in Ascomycota. The analysis revealed a majority of single presence intron positions (16 out of 39 analyzed intron positions, and up to 25 positions when single presences in a monophyletic clade were included) (Fig 3E). This finding is similar to previous reports in other eukaryotes that have experienced high rates of intron gains such as *Oikopleura dioica* and *Thalassiosira pseudonana*, in which more than 75% of intron positions are unique [26,27]. Thirteen positions show complex presence-absence patterns and a single position represents a single absence in a given species (Fig 3E). These results suggest a balanced number of putative intron gains and losses, consistent with previous intron gain and loss analyses in fungi [6,9,15,28,29].

The two ILEs that inserted in the transporter gene in *P*. *brachycarpa* are located on each side of a conserved intron in *F*. *fulva* and *D*. *septosporum* (Fig 3A). This intron is conserved in *P*. *brachycarpa* (S2 and S3 Files), suggesting that ILEs recently inserted in this gene. Orthologues of the peroxidase gene in *F*. *fulva* and *D*. *septosporum* also contain ILEs, each at different positions (Fig 3B) [14]. The occurrence of four distinct ILEs in an orthologous gene of three different fungal species suggests that this gene contains unknown features that make it very attractive for ILE insertion. All observed ILE insertions occurred at positions lacking introns in all other species (Fig 3). Clearly these ILEs are recent intron gains in *P*. *brachycarpa* and *P*. *miurae*.

Intron landscape analysis revealed six positions where intron sliding might have occurred (Fig 3). Intron sliding is a poorly understood phenomenon that refers to intron positions separated by less than 15 nucleotides [30]. It has been proposed that these positions correspond to one and the same intron that would have slightly shifted due to splice site mutations or compensatory insertion and deletion, or would have been lost and immediately reinserted [31]. Three ILEs (positions 169, 229 and 115 in Fig 3A, 3B and 3C, respectively) are separated by one, eight and three nucleotides, respectively, from a position occupied by an RSI in other fungal species (S3 File). According to the hypothesis of intron sliding, these three ILEs would be wrongly assigned as conserved ancestral introns. For each position, this hypothesis implies the ancestral insertion of an ILE that would have degenerated and, most often, been lost in all fungal species, but would not have degenerated in *P*. *brachycarpa* or *P*. *miurae*. This hypothesis strongly contradicts the observed fast degeneration of ILEs [15] and the observation that lost introns tend to be short in fungi while ILEs are long [16]. Therefore, we propose that parallel gains of two different introns are the most parsimonious explanation for these three positions. In the transporter gene, such parallel gain might have occurred very recently (Fig 3A). Indeed, although introns in *Sphaerulina* species do not share significant sequence similarity with ILEs, they are longer (81 and 87 bp; S3 File) than RSIs (50–55 bp) in fungi, and thus might represent degenerated ILEs that had inserted in the common ancestor of both *Sphaerulina* species [14].

Depending on the evolutionary model used to infer rates of intron gains, parallel gains were predicted to account for 5–10% to most of shared intron positions [10,32,33]. However, studies on genes that had been transferred from mitochondria to the nucleus revealed a very limited number of shared positions, indicating a frequency of parallel gains as low as 2.3% [34,35]. Similarly, parallel gains were estimated to contribute to only 4.2% of intron positions in *T*. *pseudonana* [26]. Our results are in striking conflict with the low number of shared positions in species that experienced high rates of intron gains as the estimated parallel gain frequency in our small dataset is 23%. However, this discrepancy likely reflects that all three ILEs inserted in positions where intron sliding might have occurred. These observations suggest that actually parallel gains might have occurred at positions where intron sliding has been proposed in Fig 3. This is particularly true for the first intron position in the oxidoreductase gene, which is occupied by an intron in *Cercospora zaeae-maydis* and in the distant species of the order *Glomerellales* (Fig 3C). In addition, the second intron position in the peroxidase gene is considered as conserved because of the occurrence of many introns within 15 nucleotides (Fig 3B). However, this position is occupied by two introns in *Lentithecium fluviatile*, suggesting that they have resulted from at least two independent gain events during the evolutionary history of *Dothideomycetes*.

## Conclusions

Our study revealed the presence of novel ILEs in eight additional non-sequenced fungal species. It suggests that ILEs are still mobile in these species where they contributed to recent intron gains. It is remarkable that amplification of novel ILEs resulted in the identification of four genes with high spliceosomal intron dynamics. The variation in intron positions in *Dothideomycetes* is consistent with ILE multiplication in this class of fungi. It was previously suggested that intron positions shared between fungi, animals and plants reflect evolutionary conservation rather than parallel gains [10,11]. However, we report three clear examples of parallel intron gains in fungi by studying intron positions of four genes only. This finding is consistent with previous reports, although mostly reported in single genes, of independent intron gains in diverse eukaryotic lineages [7,36–41]. Parallel gains were also recently reported in *Daphnia pulex* populations, in which they contributed to nearly half of all the newly discovered introns [8]. Altogether, our observations and those by others suggest that parallel intron gains might occur more frequently than previously thought in ILE-containing fungi, but also in the whole fungal kingdom and distant eukaryotes.

## Supporting Information

S1 File Sequences used to build the fungal species phylogenetic tree. (PDF)

S2 File Sequences amplified by PCR. (PDF)

S3 File DNA and amino acid alignments built to determine intron landscapes. (PDF)

S1 Table GenBank accession numbers of the ITS and LSU sequences used in this study. (PDF)

S2 Table Primers used in this study. (PDF)
