# Supplementary material for: Novel Introner-Like Elements in fungi Are Involved in Parallel Gains of Spliceosomal Introns
Source: PLoS One. 2015 Jun 5;10(6):e0129302. doi: 10.1371/journal.pone.0129302 (PMC4457414; doi:10.1371/journal.pone.0129302)
Supplement: S1 File — (PDF) [file pone.0129302.s001.pdf]

S1 File. Sequences used to build the fungal species phylogenetic tree

```
>Passalora capsicicola-CBS156.62
TAACAAGGTCTCCGTAGGTGAACCTGCGGAGGGATCATTACTGAGTGAGGGCGAAAGCCCGACCTCCAACCCTTT
GTGAACCAACTCTGTTGCTTCGGGGGCGACCCCGCCGTCTCGGCGACGGCGCCCCCGGAGGTCATCAAACACTGC
ATCTTTGCGTCGGAGTCTTGAAGTAAATTTAAACAAAACTTTCAACAACGGATCTCTTGGTTCTGGCATCGATGA
AGAACGCAGCGAAATGCGATAAGTAATGTGAATTGCAGAATTCAGTGAATCATCGAATCTTTGAACGCACATTGC
GCCCCGTGGTATTCCGCGGGGCATGCCTGTTCGAGCGTCATTTCACCACTCAAGCCTCGCTTGGTATTGGGCGTC
GCGGTTCCGCGCGCCTTAAAGTTTCCGGCTGAGCAGTTCGTCTCTAAGCGTTGTGGCATATATTTCGCTGAAGAG
CTCGGACGGTTCCGGCCGTTAAATCTTTCTAAAGGTTGACCTCGGATCAGGTAGGGATACCCGCTGAACTTAAGC
ATATGAAGCGGCAACAGCTCAAATTTGAAATCTGGCGCAAGCCCCGAGTTGTAATCTGTAGAGGATGCTTCTGGC
CAGCGGCCGTTCTAAGTTCCTTGGAACAGGACGTCATAGAGGGTGAGAATCCCGTATGTGACCGGCGCGCAGCCT
TTACGTAGCTCCTTCGACGAGTCGAGTTGTTTGGGAATGCAGCTCTAAATGGGAGGTAAATTTCTTCTAAAGCTA
AATACCGGCCAGAGACCGATAGCGCACAAGTAGAGTGATCGAAAGATGAAAAGCACTTTGGAAAGAGAGTTAAAA
AGCACGTGAAATTGTTGAAAGGGAAGCGCTTGCAACCAGACTTCGTAGCGGTGTTCCGCCGGTCTTCTGACCGGT
CTACTCATCGTCGCGAGGCCAACATCGTCTGGGACCGCTGGATAAGACCTAAGGAATGTGGCTTCCCTCGGGAAG
TGTTATAGCCTGTGGTGATGCAGCGAGTCCCGGGCGAGGTCCGCGCTTCGGCAAGGATGTTGGCGTAATGGTTGT
CAGCGGCCCGTCTTGAAACACGGACCAAGGAGTCTAACATCTATGCGAGTGTTCGGGTGTCAAACCCCTACGCGT
AATGAAAGTGAACGGAGGTGGGAAGCGCAAGCTGCACCATCGACCGATCCTGATGTCCTCGGATGGATTTGAGTA
AGAGCATAGCTGTTGGGACCCGAAAGATGGTGAACTATGCCTGAATAGGGTGAAGCCAGAGGAAACTCTGGTGGA
GGCTCGCAGCGGTTCTGACGTGCAAATCGATCGTCAAATTTGGGTATAGGGGCGAAAGACTAA
>Passalora brachycarpa-CBS115124
GTAACAAGGTCTCCGTAGGTGAACCTGCGGAGGGATCATTACTGAGTGAGGGCTCACGCCCGACCTCCAACCCTT
TGTGAACCAACTCTGTTGCTTCGGGGGCGACCCCGCCGTTTCGGCGACGGCGCCCCCGGAGGTCATCAAACACTG
CATCTTTGCGTCGGAGTCTTGAAGTAAATTTAAACAAAACTTTCAACAACGGATCTCTTGGTTCTGGCATCGATG
AAGAACGCAGCGAAATGCGATAAGTAATGTGAATTGCAGAATTCAGTGAATCATCGAATCTTTGAACGCACATTG
CGCCCCGTGGTATTCCGCGGGGCATGCCTGTTCGAGCGTCATTTCACCACTCAAGCCTAGCTTGGTATTGGGCGT
CCGCGGTTCCGCGCGCCTTAAAGTCTCCGGCTGAGCAGTTCGTCTCTAAGCGTTGTGACATATATTTCGCTGAAG
AGTTCGGACGGCTTTTGGCCGTTAAATCTTTCTCAAGGTTGACCTCGGATCAGGTAGGGATACCCGCTGAACTTA
AGCATATCAATAAGCGGAGGAAAAGAAACCAACAGGGATTGCCCTAGTAACGGCGAGTGAAGCGGCAACAGCTCA
AATTTGAAATCTGGCGCAAGCCCGAGTTGTAATTTGTAGAGGATGCTTCTGGCCAGCGGCCGTTCTAAGTTCCTT
GGAACAGGACGTCATAGAGGGTGAGAATCCCGTATGTGACCGGCGCGCAGCCTTTACGTAGCTCCTTCGACGAGT
CGAGTTGTTTGGGAATGCAGCTCTAAATGGGAGGTAAATTTCTTCTAAAGCTAAATACCGGCCAGAGACCGATAG
CGCACAAGTAGAGTGATCGAAAGATGAAAAGCACTTTGGAAAGAGAGTTAAAAAGCACGTGAAATTGTTGAAAGG
GAAGCGCTTGCAACCAGACTTCGTAGCGATGTTCCGCTGGTCTTCTGACCGGTCTACTCATCGTCGCGAGGCCAA
CATCGTCTGGGCCCGCTGGATAAGACCTAAGGAATGTGGCTTCCCTCGGGAAGTGTTATAGCCTGTGGTGATGCA
GCGTGGCTCGGGCGAGGTCCGCGCTTCGGCAAGGATGTTGGCGTAATGGTTGTCAGCGGCCCGTCTTGAAACACG
GACCAAGGAGTCTAACATCTATGCGAGTGTTCGGGTGTCAAACCCCTACGCGTAATGAAAGTGAACGGAGGTGGG
AAGCGCAAGCTGCACCATCGACCGATCCTGATGTCCTCGGATGGATTTGAGTAAGAGCATAGCTGTTGGGACCCG
AAAGATGGTGAACTATGCCTGAATAGGGTGAAGCCAGAGGAAACTCTGGTGGAGGCTCGCAGCGGTTCTGACGTG
CAAATCGATCGTCAAATTTGGGTATAGGGGCGAAAGACTAATCGAACCATCTAGTAGCTGGTTCCTGC
>Passalora smilacis-CBS556.71
GTAACAAGGTCTCCGTAGGTGAACCTGCGGAGGGATCATTACTGAGTGAGGGCTCACGCCCGACCTCCAACCCTT
TGTGAACCATCTCTGTTGCTTCGGGGGCGACCCCGCCGTCTCGGCGGCGGCGCCCCCGGAGGTCATCAAACACGG
CATCTCTGCGTCGGAGTCTTAAAGTAAATGTAAACAAAACTTTCAACAACGGATCTCTTGGTTCTGGCATCGATG
AAGAACGCAGCGAAATGCGATAAGTAATGTGAATTGCAGAATTCAGTGAATCATCGAATCTTTGAACGCACATTG
CGCCCCGTGGTATTCCGCGGGGCATGCCTGTTCGAGCGTCATTTCACCACTCAAGCCTCGCTTGGTATTGGGCGC
CGCGGTCTCCGCGCGCCTCAAAGTCTCCGGCTGGGTCGTCCGTCTCTAAGCGTTGTGGCATAGCTGTCGCTAAAG
AGTGCGGGTGGCCGTCGGCCGTTAAATCTTTCTCAAGGTTGACCTCGGATCAKGTAGGGATACCCGCTGAACTTA
AGCATATCAATAAGCGGAGGAAAAGAAACCAACAGGGATTGCCCTAGTAACGGCGAGTGAAGCGGCAACAGCTCA
AATTTGAAATCTGGCGCAAGCCCGAGTTGTAATTTGTAGAGGATGCTTCTGGCCAGCGGCCGTTCTAAGTTCCTT
GGAACAGGACGTCATAGAGGGTGAGAATCCCGTATGTGACCGGCGCGCAGCCTTTACGTAGCTCCTTCGACGAGT
CGAGTTGTTTGGGAATGCAGCTCTAAATGGGAGGTAAATTTCTTCTAAAGCTAAATACCGGCCAGAGACCGATAG
CGCACAAGTAGAGTGATCGAAAGATGAAAAGCACTTTGGAAAGAGAGTTAAAAAGCACGTGAAATTGTTGAAAGG
GAAGCGCTTGCAACCAGACTTCGCGGCGGTGTTCCGCCGGTCTTCTGACCGGTCCACTCATCGTCGCGAGGCCAA
CATCGTCTGGGCCCGCTGGATAAGACCTCAGGAATGTAGCTTCCCTCGGGAAGTGTTATAGCCCGTGGTGATGCA
GCGCGGCTCGGGCGAGGTCCGCGCTTCCGCAAGGATGTTGGCGTAATGGTTGTCAGCGGCCCGTCTTGAAACACG
GACCAAGGAGTCTAACATCTATGCGAGTGTTCGGGTGTCAAACCCCTACGCGTAATGAAAGTGAACGGAGGTGGG
AAGCGCAAGCTGCACCATCGACCGATCCTGATGTCCTCGGATGGATTTGAGTAAGAGCATAGCTGTTGGGACCCG
AAAGATGGTGAACTATGCCTGAATAGGGTGAAGCCAGAGGAAACTCTGGTGGAGGCTCGCAGCGGTTCTGACGTG
CAAATCGATCGTCAAATTTG
>Passalora miurae-CPC14643
```

TAACAAGGTCTCCGTAGGTGAACCTGCGGAGGGATCATTACTGAGTGAGGGCTCACGCCCGACCTCCAACCCTTT
GTGAACCAACTCTGTTGCTTCGGGGGCGACCCCGCCGTTTCGGCGACGGCGCCCCCGGAGGTCATCAAACACTGC
ATCTTCGCGTCGGAGTCTCAAGTAAATTTGAACAAAACTTTCAACAACGGATCTCTTGGTTCTGGCATCGATGAA
GAACGCAGCGAAATGCGATAAGTAATGTGAATTGCAGAATTCAGTGAATCATCGAATCTTTGAACGCACATTGCG
CCCCGTGGTATTCCGCGGGGCATGCCTGTTCGAGCGTCATTTCACCACTCAAGCCTAGCTTGGTATTGGGCGTCG
CGGGTCCGCGCGCCTTAAAGTCTCCGGCCGAGTAGTCCGTCTCTCAGCGTTGTGGCATATATTTCGCTGAAGAGG
TCGGGCGGCTCTTGGCCGTTAAATCTTTCTCAAGGTTGACCTCGGATCAGGTAGGGATACCCGCTGAACTTAAGC
ATATCAATAAGCGGAGGAAAAGAAACCAACAGGGATTGCCCCAGTAACGGCGAGTGAAGCGGCAACAGCTCAAAT
TTGAAATCTGGCGCAAGCCCGAGTTGTAATTTGTAGAGGATGCTTCTGGCCAGCGGCCGTTCTAAGTTCCTTGGA
ACAGGACGTCATAGAGGGTGAGAATCCCGTATGTGACCGGCGCGCAGCCTTTACGTAGCTCCTTCGACGAGTCGA
GTTGTTTGGGAATGCAGCTCTAAATGGGAGGTAAATTTCTTCTAAAGCTAAATACCGGCCAGAGACCGATAGCGC
ACAAGTAGAGTGATCGAAAGATGAAAAGCACTTTGGAAAGAGAGTTAAAAAGCACGTGAAATTGTTGAAAGGGAA
GCGCTTGCAACCAGACTTCGTAGCGATGTTCCGCTGGTCTTCTGACCGGTCTACTCATCGTCGCGAGGCCAACAT
CGTCTGGGCCCGCTGGATAAGACCTAAGGAATGTAGCTTCCCTCGGGAAGTGTTATAGCCTGTGGTGATGCAGCG
CGGCTCGGGCGAGGTCCGCGCTTCGGCAAGGATGTTGGCGTAATGGTTGTCAGCGGCCCGTCTTGAAACACGGAC
CAAGGAGTCTAACATCTATGCGAGTGTTCGGGTGTCAAACCCCTACGCGTAATGAAAGTGAACGGAGGTGGGAAG
CGCAAGCTGCACCATCGACCGATCCTGATGTCCTCGGATGGATTTGAGTAAGAGCATAGCTGTTGGGACCCGAAA
GATGGTGAACTATGCCTGAATAGGGTGAAGCCAGAGGAAACTCTGGTGGAGGCTCGCAGCGGTTCTGACGTGCAA
ATCGATCGTCAAATTTGGGTATAGGGGCGAAAGACTAATCGAACCAT
>Passalora daleae-CBS113031
TTGGAAGTAAAWRTCGTAACAAGGTCTCCGTAGGTGAACCTGCGGAGGGATCATTACTGAGTGAGGGGGCAACCC
CGACCTCCAACCCTTTGTGAACCAACTCTGTTGCTTCGGGGGCGACCCCGCCGTTTCGGCGACGGCGCCCCCGGA
GGTCATCAAACACTGCATCTATGCGTCGGAGTCTTAAAGTAAATTTAAACAAAACTTTCAACAACGGATCTCTTG
GTTCTGGCATCGATGAAGAACGCAGCGAAATGCGATAAGTAATGTGAATTGCAGAATTCAGTGAATCATCGAATC
TTTGAACGCACATTGCGCCCCGTGGTATTCCGCGGGGCATGCCTGTTCGAGCGTCATTTCACCACTCAAGCCTAG
CTTGGTATTGGGCGTCGCGGTTCCGCGCGCCTTAAAGTCTCCGGCTGAGCAGTTCGCCTCTAAGCGTTGTGGCAT
ATATTTCGCTGAAGAGTACGGACGGCTTCTGGCCGTTAAATCTTTATTCAAGGTTGACCTCGGATGCAGTAGGTC
GAGCAGCATATCAATAAGCGGAGGAAAAGAAACCCAACAGGGATTGCCCTAGTAACGGCGAGTGAAGCGGCAACA
GCTCAAATTTGAAATCTGGCGCAAGCCCGAGTTGTAATTTGTAGAGGATGCTTCTGGCCAGCGGCCGTTCTAAGT
TCCTTGGAACAGGACGTCATAGAGGGTGAGAATCCCGTATGTGACCGGCGCGCAGCCTTTACGTAGCTCCTTCGA
CGAGTCGAGTTGTTTGGGAATGCAGCTCTAAATGGGAGGTAAATTTCTTCTAAAGCTAAATACCGGCCAGAGACC
GATAGCGCACAAGTAGAGTGATCGAAAGATGAAAAGCACTTTGGAAAGAGAGTTAAAAAGCACGTGAAATTGTTG
AAAGGGAAGCGCTTGCAACCAGACTTCGTAGCGGTGTTCCGCCGGTCTTCTGACCGGTCTACTCATCGTCGCGAG
GCCAACATCGTCTGGGACCGCTGGATAAGACCTAAGGAATGTAGCTTCCCTCGGGAAGTGTTATAGCCTGTGGTG
ATGCAGCGTGTCTCGGGCGAGGTCCGCGCTTCGGCAAGGATGTTGGCGTAATGGTTGTCAGCGGCCCGTCTTGAA
ACACGGACCAAGGAGTCTAACATCTATGCGAGTGTTCGGGTGTCAAACCCCTACGCGTAATGAAAGTGAACGGAG
GTGGGAAGCGCAAGCTGCACCATCGACCGATCCTGATGTCCTCGGATGGATTTGAGTAAGAGCATAGCTGTTGGG
ACCCGAAAGATGGTGAACTATGCCTGAATAGGGTGAAGCCAGAGGAAACTCTGGTGGAGGCTCGCAGCGGTTCTG
ACGTGCAAATCGATCGTCAAATTTGGGTATAGGGGCGAAAGACTAATCGAACCATCTAGTAGCTGGTTCCTGCCG
AAG
>Passalora perfoliati-CBS113378
TAACAAGGTCTCCGTAGGTGAACCTGCGGAGGGATCATTACTGAGTGAGGGCGCAAGCCCGACCTCCAACCCTTT
GTGAACCAACTCTGTTGCTTCGGGGGCGACCCCGCCGTTTCGGCGACGGCGCCCCCGGAGGTCCTCAAACACTGC
ATCTTTGCGTCGGAGTCTTAAAGTAAATTTAAACAAAACTTTCAACAACGGATCTCTTGGTTCTGGCATCGATGA
AGAACGCAGCGAAATGCGATAAGTAATGTGAATTGCAGAATTCAGTGAATCATCGAATCTTTGAACGCACATTGC
GCCCCGTGGTATTCCGCGGGGCATGCCTGTTCGAGCGTCATTTCACCACTCAAGCCTCGCTTGGTATTGGGCGTC
GCGGTTCCGCGCGCCTTAAAGTCTCCGGCTGAGCAGTTACGTCTCTAAGCGTTGTGGCATATCAATTCGCTGAAG
AGTTTCGGACGGCTTTTGGCCGTTAAATCTTTCTTAAGGTTGACCTCGGATCAGGTAGGGATACCCGCTGAACTT
AAGCATATCAAGAAGCGGCAACAGCTCAAATTTGAAATCTGGCGCAAGCCCCGAGTTGTAATTTGTAGAGGATGC
TTCTGGCCAGCGGGCCGTTCTAAGTTCCTTGGAACAGGACGTCATAGAGGGTGAGAATCCCGTATGTGACCGGCG
CGCAGCCTTTACGTAGCTCCTTCGACGAGTCGAGTTGTTTGGGAATGCAGCTCTAAATGGGAGGTAAATTTCTTC
TAAAGCTAAATACCGGCCAGAGACCGATAGCGCACAAGTAGAGTGATCGAAAGATGAAAAGCACTTTGGAAAGAG
AGTTAAAAAGCACGTGAAATTGTTGAAAGGGAAGCGCTTGCAACCAGACTTCGTAGCGATGTTCCGCCGGTCTTC
TGACCGGTCTACTCATCGTCGCGAGGCCAACATCGTCTGGGACCGCTGGATAAGACCTAAGGAATGTAGCTTCCC
TCGGGAAGTGTTATAGCCTGTGGTGATGCGGCGTGTCTCGGGCGAGGTCCGCGCTTCGGCAAGGATGTTGGCGTA
ATGGTTGTCAGCGGCCCGTCTTGAAACACGGACCAAGGAGTCTAACATCTATGCGAGTGTTCGGGTGTCAAACCC
CTACGCGTAATGAAAGTGAACGGAGGTGGGAAGCGCAAGCTGCACCATCGACCGATCCTGATGTCCTCGGATGGA
TTTGAGTAAGAGCATAGCTGTTGGGACCCGAAAGATGGTGAACTATGCCTGAATAGGGGTGAAGCCAGAGGAAAA
CTCTGGTGGAGGCTCGCAGCGGTTCTGACGTGCAAATCGATCGTCAAATTTGGGTATAGGGGCGAAAGACT
>Amycosphaerella africana-CBS116154
CTGCGGAGGGATCATTACTGAGTGAGGGCTCACGCCCGACCTCCAACCCTTTGTGAACCAACTCTGTTGCTTCGG
GGGCGACCCCGCCGTTTCGGCGACGGCGCCCCCGGAGGTCATCAAACACTGCATCTTTGCGTCGGAGTCTTAAAG
TAAATTTAAACAAAACTTTCAACAACGGATCTCTTGGTTCTGGCATCGATGAAGAACGCAGCGAAATGCGATAAG

TAATGTGAATTGCAGAATTCAGTGAATCATCGAATCTTTGAACGCACATTGCGCCCCGTGGTATTCCGCGGGGCA
TGCCTGTTCGAGCGTCATTTCACCACTCAAGCCTAGCTTGGTATTGGGCGTCGCGGTTCCGCGCGCCTTAAAGTC
TCCGGCTGAGCAGTTCGTCTCTAAGCGTTGTGGCATATATTTCGCTGAAGAGTTCGGACGGCTTTTGGCCGTTAA
ATCTTTCTTAAGGTTGACCTCGGATCAGGTAGGGATACCCGCTGAACTTAAGCATATCAAGGAAAAGAAACCAAC
AGGGATTGCCCTAGTAACGGCGAGTGAAGCGGCAACAGCTCAAATTTGAAATCTGGCGCAAGCCCGAGTTGTAAT
TTGTAGAGGATGCTTCTGGCCAGCGGCCGTTCTAAGTTCCTTGGAACAGGACGTCATAGAGGGTGAGAATCCCGT
ATGTGACCGGCGCGCAGCCTTTACGTAGCTCCTTCGACGAGTCGAGTTGTTTGGGAATGCAGCTCTAAATGGGAG
GTAAATTTCTTCTAAAGCTAAATACCGGCCAGAGACCGATAGCGCACAAGTAGAGTGATCGAAAGATGAAAAGCA
CTTTGGAAAGAGAGTTAAAAAGCACGTGAAATTGTTGAAAGGGAAGCGCTTGCAACCAGACTTCGTAGCGATGTT
CCGCTGGTCTTCTGACCGGTCTACTCATCGTCGCGAGGCCAACATCGTCTGGGCCCGCTGGATAAGACCTAAGGA
ATGTGGCTTCTCTTCGGGGAAGTGTTATAGCCTGTGGTGATGCAGCGTGGCTCGGGCGAGGTCCGCGCTTCGGCA
AGGATGTTGGCGTAATGGTTGTCAGCGGCCCGTCTTGAAACACGGACCAAGGAGTCTAACATCTATGCGAGTGTT
CGGGTGTCAAACCCCTACGCGTAATGAAAGTGAACGGAGGTGGGAAGCGCAAGCTGCACCATCGACCGATCCTGA
TGTCCTCGGATGGATTTGAGTAAGAGCATAGCTGTTGGGACCCGAAAGATGGTGAACTATGCCTGAATAGGGTGA
AGCCAGAGGAAACTCTGGTGGAGGCTCGCAGCGGTTCTGACGTGCAAATCGATCGTCAAATTTGGGTATAGGGGC
GAAAGACTAATCGAACCATC
>Passalora microsora-CBS123735
TAACAAGGTCTCCGTAGGTGAACCTGCGGAGGGATCATTACTGAGTGAGGGCGCAAGCCCGACCTCCAACCCTTT
GTGAACCAACTCTGTTGCTTCGGGGGCGACCCCGCCGTTTCGGCGACGGCGCCCCCGGAGGTCATCAAACACTGC
ATCTTTGCGTCGGAGTCTTAAAGTAAATTTAAACAAAACTTTCAACAACGGATCTCTTGGTTCTGGCATCGATGA
AGAACGCAGCGAAATGCGATAAGTAATGTGAATTGCAGAATTCAGTGAATCATCGAATCTTTGAACGCACATTGC
GCCCCGTGGTATTCCGCGGGGCATGCCTGTTCGAGCGTCATTTCACCACTCAAGCCTAGCTTGGTATTGGGCGTC
GCGGTTCCGCGCGCCTTAAAGTCTCCGGCTGAGCAGTTCGTCTCTAAGCGTTGTGACATATATTTCGCTGAAGAG
TTCGGGCGGCTTTTGGCCGTTAAATCTTTCTTAAGGTTGACCTCGCGAGTGAAGCGGCAACAGCTCAAATTTGAA
ATCTGGCGCAAGCCCGAGTTGTAATTTGTAGAGGATGCTTCTGGCCCAGCGGCCGTTCTAAGTTCCTTGGAACAG
GACGTCATAGAGGGTGAGAATCCCGTATGTGACCGGCGCGCAGCCTTTACGTAGCTCCTTCGACGAGTCGAGTTG
TTTGGGAATGCAGCTCTAAATGGGAGGTAAATTTCTTCTAAAGCTAAATACCGGCCAGAGACCGATAGCGCACAA
GTAGAGTGATCGAAAGATGAAAAGCACTTTGGAAAGAGAGTTAAAAAGCACGTGAAATTGTTGAAAGGGAAGCGC
TTGCAACCAGACTTCGTAGCGATGTTCCGCTGGTCTTTTGACCGGTCTACTCATCGTCGCGAGGCCAACATCGTC
TGGGCCCGCTGGATAAGACCTAAGGAATGTAGCTTCCCTCGGGAAGTGTTATAGCCTGTGGTGATGCAGCGCGGC
TCGGGCGAGGTCCGCGCTTCGGCAAGGATGTTGGCGTAATGGTTGTCAGCGGCCCGTCTTGAAACACGGACCAAG
GAGTCTAACATCTATGCGAGTGTTCGGGTGTCAAACCCCTACGCGTAATGAAAGTGAACGGAGGTGGGAAGCGCA
AGCTGCACCATCGACCGATCCTGATGTCCTCGGATGGATTTGAGTAAGAGCATAGCTGTTGGGACCCGAAAGATG
GTGAACTATGCCTGAATAGGGTGAAGCCAGAGGAAAACTCTGGTGGAGGCTCGCAGCGGTTCTGACGTGCAAATC
GATCGTCAAATTTGGGTATAGGGGCGAAAGACTAATC
>Fulvia fulva-CBS131901
TTTAGAGGAAGTAAAAGTCGTAACAAGGTCTCCGTAGGTGAACCTGCGGAGGGATCATTACTGAGTGAGGGCTCA
CGCCCGACCTCCAACCCTCTGTGAACCAACTCTGTTGCTTCGGGGGCGACCCCGCCGTTTCGGCGACGGCGCCCC
CGGAGATCATCAAACACTGCATCTTTGCGTCGGAGTCTTAAAGTAAATTTAAACAAAACTTTCAACAACGGATCT
CTTGGTTCTGGCATCGATGAAGAACGCAGCGAAATGCGATAAGTAATGTGAATTGCAGAATTCAGTGAATCATCG
AATCTTTGAACGCACATTGCGCCCCGTGGTATTCCGCGGGGCATGCCTGTTCGAGCGTCATTTCACCACTCAACG
CCTAGCTTGGTATTGGGCGTCGCGGTTCCGCGCGCCTTGAACGTCTCCGGCTGAGCAGTTCGTCTCTAAGCGTTG
TGGCATATATTTCGCTAAAGAGTTCGGGCGGCTTTTGGCCGTTAAATCTTTTCAAAGGTTGACCTCGGATCAGGT
AGGGATACCCGCTGAACTTAAGCATATCAATAAGCGGAGGAAAAGAAACCAACAGGGATTGCCCTAGTAACGGCG
AGTGAAGCGGCAACAGCTCAAATTTGAAATCTGGCGCAAGCCCGAGTTGTAATTTGTAGAGGATGCTTCTGGCCA
GCGGCCGTTCTAAGTTCCTTGGAACAGGACGTCATAGAGGGTGAGAATCCCGTATGTGACCGGTGCGCAGCCTTT
ACGTAGCTCCTTCGACGAGTCGAGTTGTTTGGGAATGCAGCTCTAAATGGGAGGTAAATTTCTTCTAAAGCTAAA
TACCGGCCAGAGACCGATAGCGCACAAGTAGAGTGATCGAAAGATGAAAAGCACTTTGGAAAGAGAGTTAAAAAG
CACGTGAAATTGTTGAAAGGGAAGCGCTTGCAACCAGACTTCGTAGCGATGTTCCGCTGGTCTTCTGACCGGTCT
ACTCATCGTCGCGAGGCCAACATCGTCTGGGACCGCTGGATAAGACCTAAGGAATGTAGCTTCCCGTCGGGAAGT
GTTATAGCCTGTGGTGATGCAGCGCGTCGTCGGGCGAGGTCCGCGCTTCGGCAAGGATGTTGGCGTAATGGTTGC
CAGCGGCCCGTCTGAAACACGGACCAAGGAGTCTAACATCTATGCGAGTGTTCGGGTGTCAAACCCCTACGCGTA
ATGAAAGTGAACGGAGGTGGGAAGCGCAAGCTGCACCATCGACCGATCCTGATGTCCTCGGATGGATTTGAGTAA
GAGCATAGCTGTTGGGACCCGAAAGATGGTGAACTATGCCTGAATAGGGTGAAGCCAGAGGAAACTCTGGTGGAG
GCTCGCAGCGGTTCTGACGTGCAAATCGATCGTCAAATTTGGGTATAGGGGCGAAAGACTAATCGAACCATCTAG
TAGCTGGTTCCTGCCGAAG
>Dothistroma septosporum-CBS128990
TTTAGAGGAAGTAAAAGTCGTAACAAGGTCTCCGTAGGTGAACCTGCGGAGGGATCATTACTGAGTGAGGGCGAA
AGCCCGACCTCCAACCCTTTGTGAACCAACTCTGTTGCTTCGGGGGCGACCCCGCCGTTTCGGCGACGGCGCCCC
CGGAGGTCATCAAACACTGCATCTTTGCGTCGGAGTCTTAAAGTAAATTTAAACAAAACTTTCAACAACGGATCT
CTTGGTTCTGGCATCGATGAAGAACGCAGCGAAATGCGATAAGTAATGTGAATTGCAGAATTCAGTGAATCATCG
AATCTTTGAACGCACATTGCGCCCCGTGGTATTCCGCGGGGCATGCCTGTTCGAGCGTCATTTCACCACTCAAGC
CTGGCTTGGTATTGGGCGTCGCGGTTCCGCGCGCCTTAAAGTCTCCGGCTGAGCAGTTCGTCTCTAAGCGTTGTG

GCATATATTTCGCTGAAGAGTTCGGACGGCTTTTGGCCGTTAAATCTTTTACAAGGTTGACCTCGGATCAGGTAG
GGATACCCGCTGAACTTAAGCATATCAATAAGCGGAGGAAAAGAAACCAACAGGGATTGCCCTAGTAACGGCGAG
TGAAGCGGCAACAGCTCAAATTTGAAATCTGGCGCAAGCCCGAGTTGTAATTTGTAGAGGATGCTTCTGGCCAGC
GGCCGTTCTAAGTTCCTTGGAACAGGACGTCATAGAGGGTGAGAATCCCGTATGTGACCGGCGCGCAGCCTTTAC
GTAGCTCCTTCGACGAGTCGAGTTGTTTGGGAATGCAGCTCTAAATGGGAGGTAAATTTCTTCTAAAGCTAAATA
CCGGCCAGAGACCGATAGCGCACAAGTAGAGTGATCGAAAGATGAAAAGCACTTTGGAAAGAGAGTTAAAAAGCA
CGTGAAATTGTTGAAAGGGAAGCGCTTGCAACCAGACTTCGTAGCGATGTTCCGCCGGTCTTCTGACCGGTCTAC
TCATCGTCGCGAGGCCAACATCGTCTGGGGCCGCTGGATAAGACCTAAGGAATGTAGCTTCCCTCGGGAAGTGTT
ATAGCCTGTGGTGATGCAGCGTGTCTCGGGCGAGGTCCGCGCTTCGGCAAGGATGTTGGCGTAATGGTTGTCAGC
GGCCCGTCTTGAAACACGGACCAAGGAGTCTAACATCTATGCGAGTGTTCGGGTGTCAAACCCCTACGCGTAATG
AAAGTGAACGGAGGTGGGAAGCGCAAGCTGCACCATCGACCGATCCTGATGTCCTCGGATGGATTTGAGTAAGAG
CATAGCTGTTGGGACCCGAAAGATGGTGAACTATGCCTGAATAGGGTGAAGCCAGAGGAAACTCTGGTGGAGGCT
CGCAGCGGTTCTGACGTGCAAATCGATCGTCAAATTTGGGTATAGGGGCGAAAGACTAATCGAACCATCTAGTAG
CTGGTTCCTGCCGAAG
>Pseudocercospora fijiensis-CIRAD86
TTTAGAGGAAGTAAAAGTCGTAACAAGGTCTCCGTAGGTGAACCTGCGGAGGGATCATTACCGAGTGAGGGCTCA
CGCCCGACCTCCAACCCTTTGTGAACCACAACTTGTTGCTTCGGGGGCGACCTGCCGTCGGCGGGCGCCCCCGGA
GGCCGTCTAAACACTGCATCTTTGCGTCGGAGTTTCAAACAAATCGAACAAAACTTTCAACAACGGATCTCTTGG
TTCTGGCATCGATGAAGAACGCAGCGAAATGCGATAAGTAATGTGAATTGCAGAATTCAGTGAATCATCGAATCT
TTGAACGCACATTGCGCCCTTTGGTATTCCGAAGGGCATGCCTGTTCGAGCGTCATTTCACCACTCAAGCCTGGC
TTGGTATTGGGCGTCGCGGTTCTTCGCGCGCCTTAAAGTCTCCGGCTGAGCTGTCCGTCTCTAAGCGTTGTGGAT
CTTTCAATTCGCTTCGGAGTGCGGGTGGCCGCGGCCGTTAAATCTTTATTCAAAGGTTGACCTCGGATCAGGTAG
GGATACCCGCTGAACTTAAGCATATCAATAAGCGGAGGAAAAGAAACCAACAGGGATTGCCCTAGTAACGGCGAG
TGAAGCGGCAACAGCTCAAATTTGAAATCTGGCGTAAGCCCGAGTTGTAATTTGTAGAGGATGCTTCGGGGTAGC
GGCCGGTCTAAGTTCCTTGGAACAGGACGTCATAGAGGGTGAGAATCCCGTACGTGACTGGCTTGCACCCTCCAC
GTAGCTCCTTCGACGAGTCGAGTTGTTTGGGAATGCAGCTCTAAATGGGAGGTAAATTTCTTCTAAAGCTAAATA
CCGGCCAGAGACCGATAGCGCACAAGTAGAGTGATCGAAAGATGAAAAGCACTTTGGAAAGAGAGTTAAAAAGCA
CGTGAAATTGTTGAAAGGGAAGCGCCCGCAACCAGACTTTGCGGCGGTGTTCGGCCGGTCTTCTGACCGGTTTAC
TCGCCGCCGTGAGGCCATCATCGTCTGGGACCGCTGGATAAGACCCGAGGAATGTAGCTCCCTTCGGGGTGTGTT
ATAGCCTCGGGTGATGCAGCGCGTCCCGGGCGAGGTCCGCGCTTCGGCAAGGATGATGGCGTAATGGTTGTCGGC
GGCCCGTCTTGAAACACGGACCAAGGAGTCTAACATCTATGCGAGTGTTCGGGTGTCAAACCCCTACGCGTAATG
AAAGTGAACGGAGGTGGGAACTTTTGGTGCACCATCGACCGATCCTGATGTCCTCGGATGGATTTGAGTAAGAGC
ATAGCTGTTGGGACCCGAAAGATGGTGAACTATGCCTGAATAGGGTGAAGCCAGAGGAAACTCTGGTGGAGGCTC
GCAGCTATAATATATTAAAATCGAACCTAGCGAGATAGACGGCCTATCTAGCCTATCGACCTATAACCCTTATAA
CCTTAGTA
>Zymoseptoria tritici-IPO323
TTTAGAGGAAGTAAAAGTCGTAACAAGGTCTCCGTAGGTGAACCTGCGGAGGGATCATTACCGAGCGAGGGCCTC
CGGGTCCGACCTCCAACCCTTTGTGAACACATCCCGTTGCTTCGGGGGCGACCCTGCCGGGCGCCCCCGGAGGAC
CACCAAAAAACACTGCATCTCTGCGTCGGAGTTTACGAGTAAATCGAAACAAAACTTTCAACAACGGATCTCTTG
GTTCTGGCATCGATGAAGAACGCAGCGAAATGCGATAAGTAATGTGAATTGCAGAATTCAGTGAATCATCGAATC
TTTGAACGCACATTGCGCCCCCTGGTATTCCGGGGGGCATGCCCGTTCGAGCGTCATTACACCACTCCAGCCTCG
CTGGGTATTGGGCGTCTTTTCGCGGGGGATCACTCCCCCGCGCGCCTCAAAGTCTCCGGCTGAGCGGTCTCGTCT
CCCAGCGTTGTGGCATCACGTCTCGCCGCGGAGTTCACGAGCCCTCACGGCCGTTAAATCACACCTCAGGTTGAC
CTCGGATCGGGTAGGGATACCCGCTGAACTTAAGCATATCAATAAGCGGAGGAAAAGAAACCAACAGGGATTGCC
CTAGTAACGGCGAGTGAAGCGGCAACAGCTCAAATTTGAAATCTGGCCCCCCGGCCCGAGTTGTAATTTGTAGAG
GATGCTTCTGGGTAGCGACCGGTCTAAGTTCCTTGGAACAGGACGTCATAGAGGGTGAGAATCCCGTATGCGACC
GGCCCGCGCCCTCCACGTAGCTCCTTCGACGAGTCGAGTTGTTTGGGAATGCAGCTCTAAATGGGAGGTAAATTT
CTTCTAAAGCTAAATACCGGCCAGAGACCGATAGCGCACAAGTAGAGTGATCGAAAGATGAAAAGCACTTTGGAA
AGAGAGTTAAAAAGCACGTGAAATTGTTGAAAGGGAAGCGCTTACAACCAGACTTTGGGGCGGTGTTCCGCCGGT
CTTCTGACCGGTCTACTCTCCGTCCCGAGGCCAACATCATCTGGGACCGCCGGACAAGACCTCAGGAATGTAGCT
CCCCCTCGGGGGAGTGTTATAGCCTGTGGTGATGCGGCGCGTCCCGGGTGAGGTCCGCGCTTCGGCAAGGATGTT
GGCGTAATGGTTGTCAGCGGCCCGTCTTGAAACACGGACCAAGGAGTCTAACATCTATGCGAGTGTTCGGGTGTC
AAACCCCTACGCGGAATGAAAGTGAACGGAGGTGGGAAGGGGCAACCCTGCACCATCGACCGATCCTGATGTCCT
CGGATGGATTTGAGTAAGAGCATAGCTGTTGGGACCCGAAAGATGGTGAACTATGCCTGAATAGGGTGAAGCCAG
AGGAAACTCTGGTGGAGGCTCGCAGCGGTTCTGACGTGCAAATCGATCGTCAAATTTGGGTATAGGGGCGAAAGA
CTAATCGAACCATCTAGTAGCTGGTTCCTGCCGAAG
>Aspergillus niger-CBS513.88
TTTAGAGGAAGTAAAAGTCGTAACAAGGTTTCCGTAGGTGAACCTGCGGAAGGATCATTACCGAGTGCGGGTCCT
TTGGGCCCAACCTCCCATCCGTGTCTATTGTACCCTGTTGCTTCGGCGGGCCCGCCGCTTGTcggccgccggggg
ggcgcctctgccccccgggcccgtgcccgccggAGACCCCAACACGAACACTGTCTGAAAGCGTGCAGTCTGAGT
TGATTGAATGCAATCAGTTAAAACTTTCAACAATGGATCTCTTGGTTCCGGCATCGATGAAGAACGCAGCGAAAT
GCGATAACTAATGTGAATTGCAGAATTCAGTGAATCATCGAGTCTTTGAACGCACATTGCGCCCCCTGGTATTCC
GGGGGGCATGCCTGTCCGAGCGTCATTGCTGCCCTCAAGCCCGGCTTGTGTGTTGGGTCGCCGTCCCCCTCTCCG

GGGGGACGGGCCCGAAAGGCAGCGGCGGCACCGCGTCCGATCCTCGAGCGTATGGGGCTTTGTCACATGCTCTGT
AGGATTGGCCGGCGCCTGCCGACGTTTTCCAACCATTCTTTCCAGGTTGACCTCGGATCAGGTAGGGATACCCGC
TGAACTTAAGCATATCAATAAGCGGAGGAAAAGAAACCAACCGGGATTGCCTCAGTAACGGCGAGTGAAGCGGCA
AGAGCTCAAATTTGAAAGCTGGCTCCTTCGGAGTCCGCATTGTAATTTGCAGAGGATGCTTTGGGTGCGGCCCCC
GTCTAAGTGCCCTGGAACGGGCCGTCAGAGAGGGTGAGAATCCCGTCTTGGGCGGGGTGTCCGTGCCCGTGTAAA
GCTCCTTCGACGAGTCGAGTTGTTTGGGAATGCAGCTCTAAATGGGTGGTAAATTTCATCTAAAGCTAAATACTG
GCCGGAGACCGATAGCGCACAAGTAGAGTGATCGAAAGATGAAAAGCACTTTGAAAAGAGAGTTAAACAGCACGT
GAAATTGTTGAAAGGGAAGCGCTTGCGACCAGACTCGCCCGCGGGGTTCAGCCGGCATTCGTGCCGGTGTACTTC
CCCGTGGGCGGGCCAGCGTCGGTTTGGGCGGCCGGTCAAAGGCCCCTGGAATGTAGTGCCCTCCGGGGCACCTTA
TAGCCAGGGGTGCAATGCGGCCAGCCTGGACCGAGGAACGCGCTTCGGCACGGACGCTGGCATAATGGTCGTAAA
CGACCCGTCTTGAAACACGGACCAAGGAGTCTAACATCTACGCGAGTGTTCGGGTGTCAAACCCGTGCGCGCAGT
GAAAGCGAACGGAGGTGGGAGCCCCCTTGCGGGGCGCACCATCGACCGATCCTGATGTCTTCGGATGGATTTGAG
TAAGAGCGTAGCTGTGGGGACCCGAAAGATGGTGAACTATGCCTGAATAGGGCGAAGCCAGAGGAAACTCTGGTG
GAGGCTCGCAGCGGTTCTGACGTGCAAATCGATCGTCAAATTTGGGTATAGGGGCGAAAGACTAATCGAACCATC
TAGTAGCTGGTTCCTGCCGAAG
