# Supplementary material for: Novel Introner-Like Elements in fungi Are Involved in Parallel Gains of Spliceosomal Introns
Source: PLoS One. 2015 Jun 5;10(6):e0129302. doi: 10.1371/journal.pone.0129302 (PMC4457414; doi:10.1371/journal.pone.0129302)
Supplement: S2 File — (PDF) [file pone.0129302.s002.pdf]

S2 File. Sequences amplified by PCR.

Oligonucleotide sequences are highlighted in yellow. When two several introns were amplified, they are underlined. The presence of AA in the sequence name indicates that the PCR fragment was cloned before sequencing.

>Fulful-cf01-AA30 (*cf01019*)
CTGAAGGGTGCTAGGCATCTTCGCGTGTCTGAGACAACTGCTGACCTTGTACCCTTTGCTTTT
>Fulful-cf01-AA31(cf01001-02-03-04-05-11-22)
CTGAAGGGTGCTAGGCATCTTCGCGTGTCTGAGACATCTGCTGACCTTGTACCCTTTGCTTTT
>Pasmiu-cf01-AA32 (inserted in Fulfu186212)
AGGGTGCTAGGCATCTTCGCTTGTCTCAGGCATCTGATAACCTTGGACCCTTTGCTTTTAGGTATAGTGGCGAGA
ACATGCGTCTGGAGACGATCGGTCTACTGTTCACAACAACAGCCACTGCATGCCTTCTTGGTTTGGCACGCGATG
ATGATAAACACACGGAATTTGTAGAAGCGATGTATCGAGGCAGCACAACCTGCCTACACCTCGTAAGTAAATCGG
AGGGTTCTAGGCACCTTTACATTTCTCAGACACCTACTGACCTTGTACCCTTTGCTTTT
>Pasmiu-cf01-AA33
CTGAAGGGTGCTAGGCATCTTCGCGTGTCTGAGACATCTGCTGACCTGGTACCCTTTGCTTTT
>Passmi-cf01-AA35
CTGAAGGGTGCTAGGCATCTTCGCTTGCCTTAGATATCTGCTGACCTTGTACCCTTTGCTTTT
>Pasmic-cf01-AA36
CTGAAGGGTGCTAGGCATCTTCGCTCGCTCGCCTGTCTAAGAAGACATCCGCTGACCTTGTACCCTTTGCTTTT
>Pasmic-cf01-AA37
CTGAAGGGTGCTAGGCATCTTCGCCTGTCTAAGACATCCGCTGACCTTGTACCCTTTGCTTTT
>Pasbra-cf01-AA40
CTGAAGGGTGCTAGGCATCTTCGAGTGTCTCACACGTTTGCTAACCTTGTACCCTTTGCTTTT
>Pasper-cf01-AA42
CTGAAGGGTGCTAGGCATCTTCGCTTGTCTTAGACACCTACTGACCTTGTACCCTTTGCTTTT
>Pasper-cf01-AA43
CTGAAGGGTGCTAGGCATCTTCGCTTGTCTTAGACATCTACTGACCTTGTACCCTTTGCTTTT
>Zymtri-cf01-AA44 (Zymtr43394 from 5'UTR to 2nd intron, *mg01020*)
CTGAAGGGTGCTAGGCATCTTCGAACTTACCTTGCCTGCCTCATCTACCTCCACGTCCATCTTCCAGCACTCAAC
TTCGCAGCTGGAGTCTTCACCATACCAAACATGTTCTTCTCCAAATTCGTTTCCATCGCGACGGTGTTGGTCGCC
AGCGGGCAAGCTCTCCCAAGCTTGGAAGATCGCCAGTCCGGCTGTCCGCCAATCCACATCTTCGGCGCGCGCGAG
ACGACAGCATCGCCAGGCTTTGGAACCGCAGGCGTCTTCATCAATCTCATCGTAGGCGCATATCCCTCGGCCACC
ACGGAGGCGATCAACTATCCAGCCACTGGTGGAGACTCTTATGGCAGTTCAATGAGGACTGGTGTGGCCAACATC
GCCAACCAGATCAATTCTTTCAACCAGAGATGCCCTCAGGCAAAGCTCGTCGTCGTCGGGTATTCGCAGGTATGT
CCTTTGGCAGAGGTGGTGTGTGTGTGTGCTTCTCATCCATCAGAGCTGACGATATCCAGGGCGCGCAAATCTCCG
ACAATGCCATCTGCGGCGGCGGAGACCCGAATCAAGGTAAGAAACCGAAGTGCAGAAGGCCTCCATTGTCCAAGA
CTGTCTCGGACGCCAGGCTTCCATCCATGCTTCGAAGTCTACGACATCCGCTGACCTTGTACCCTTTGCTTTT
>Zymtri-cf01-AA45 (*mg01022*)
CTGAAGGGTGCTAGGCATCTTCGCGTGTCTGAGACATCTGCTGACCTTGTACCCTTTGCTTTT
>Pasbra-cf01-11-7-2 (inserted in Clafu193200)
CTGAAGGGTGCTAGGCATCTTCGCATGTCTCAGACGTATACTGATGTTGATTGCCTTGCTTTTAGTCATTCTCGG
AAAGTTCATTATTGCTGCGGTCGCGGTCACGAACGGCTACGTAGGTGCTGAGTGGCATATCGGGTTTCCAGTCGT
TAGCAGATATGTGTGGGGCATCTATGGGTTCGATCACCTCATCACATGCTTGCAACCAGCAGACTGACCTAGTGG
CACAGACACTACGTTGCCCTGGTGCAGCGTGTGATACTTTCTCTGGTATGGTTCGCAGTACAGTCCTGGACGGGC
GGTCTTTGCGTTCAGAACATCTTGGCTGCCATCTTCCCTTCGTACCAGAACATGGGCGACGTCTTCCCGGCCTCG
GCCAACATGAATGCCAAACAATGTAAGTAAACCGAAGGGTTCTGGCCACCCTCGAATGTCCGAGACATCTGCTGA
CCTTGTACCCTTTGCTTTTAG
>Fulful-cf0203-AA57 (*cf02001*)
GGGCGGAAGGCATCATCTGTCTGGAACATGCGCGCAGCATCCCTAGCTTAGTCGCGGTTGCTGAGACAATGCCAC
GTGGTGTTCGAGTATGGCTAACACCCTTCTGTTTC
>Fulful-cf0203-AA58 (*cf02015* + downstream RSI)
GGGCGGAAGGCATCATCTGTCTGACACATGCGCGCGGCATCCCCAGCTTAGTCGCGTTTGCTGAGACATTGCCAC
GTGGTGTTCGAGTATGACTGACACCCTTCTGTTTAGGTTTCACTTTCTAGTAAGGGCCTCGTTCACTACAGTCCC
AGACACTGCCATGTGGTTCTCGAGTATAGCTAACACCCTTCTGTTTC
>Fulful-cf0203-AA59-REV (*cf02004*)
GGGTGGAAGGCATCATCTGTCTGGAACATGCGCGCGGCATCCCTAGCTTAGTCGCGGTTGCTGAGACAATGCCAC
GTGGTGTTCGAGTATGGCTAACACCCTTCTGTTTC
>Pasmiu-cf0203-AA60-REV

GGGTGGAAGGCATCGTCTCTATGAGACATGCGCACAGCACCTCCACTTTATTGGCACTTGCTGAGACACTGCCAC
GTGGTTCTCGAGCATGGCTAACACCCTTCTGTTTC
>Pasmiu-cf0203-AA61-REV (inserted in Zymtr43851)
GGGCGGAAGGCATCGTCTGTTCACGATCGTCCGCGCCTCACTCCCTGCTGGACATCAGTGGATGAGACATCGCCA
CATGCTGCTCGAGTATAGCTAATGCTTTTCTTCGGTAGTCGCCAAGACCTCCATGACTGTTTGAAGCAGGTCGCC
ACTTCGTCGGATCGGCCTGGTCCGCCAGCTGTTGTCCGCGTTTTCAGCAAAGTTGTGTCTTGCCAATGCGAGGCT
GGGATCGTCCAGCTGGACAACGGCGAGGAACTTGAGGCAGACCTCATTGTCGGAGCGGATGGTATCCACAGCGTT
CTGCGGGACTGCGTCTCTTCAGAAGGCTCCAAGCCGATCCCGACAGGCCTCTCTGCCTATCGACTAATGTTGCCA
AGCGCGGTCATTCAAAGGGACGCACCGCAGTTCTGTGCCAACGTCGATCCACGAGAACCCTACACCTCAATGATC
ATGGCGCACCAATGCCGTCTGATCATGGGACCGGCTAGAAACGGCGAGCTCTATAGTCTTGTGGGCTTGGTACCA
GATGACCAGATGAATGAAGATCCGAACAGCAAACAATCGTGGGTGTCGAAGGGGAGCATCAACAAAATGCTTGAC
ACTTACCAAGAGTTTCCCCACTGGATAAAGTCACTCCTTACGGCCGCAAATCAACAAGACATTGGACTGTGGCAA
CTTCGAGACATTGAGCCCTTGACGACCTGGTATAAAGGCAAGGTTATTCTCATCGGCGACGCTGGTAAGTGCGAA
GCGTGGGCAGCATTGGCTGGAATACGCGCGCTACCCTCGCGCTCAGCACGAGCTGCTGAGACCCTGTCCGTTCCT
GCTCGAACATAGCTAACACCCTTCTGTTTC
>Passmi-cf0203-AA62-REV
GGGTGGAAGGCATCGTCTGTCTTATACGTGCCCGCGGCTCCCCCCCCTCTTTAGCACCTCCAGCGGAGACATTGC
CACGTAGTGTTCGAGTATGGCTAACACCCTTCTGTTTC
>Pasmic-cf0203-AA63
GGGCGGAAGGCATCGTCTGTCTTAGACGTGCCCGAAGGCTCACTCCCGTCTCAGCACCACCAGCTGAGACACTGC
CACGTGGTGTTCGAGTATGGCTAACACCCTTCTGTTTC
>Amyafr-cf0203-AA65
GGGCGGAAGGCATCGTCTGTCTGAGACCTGCACGCGGCATCTCCAGCTCAGCAGTAGTTGCTAAGACATTGCTAC
GTGGTGTTCGAGTATGGCTAACACCCTTCTGTTTC
>Pasbra-cf0203-AA66-REV
GGGTAGAAGGCATCGTCTGTCTGAGACATGCGCGCGGCATCTCCAGCTCAATAGCCACTGCTGAGACATTGCCAC
GTGGAGTTCGAGTATGGCTAACACCCTTCTGTTTC
>Pasdal-cf0203-AA67-REV
GGGCGGAAGGCATCGTCTAACCAAGAAATGCGCGCCGCATCTCCGACTCGTCAGCAGCTGCTGATACATTGCCAC
GTGGTTCTCGAGTATAGCTAACACCCTTCTGTTTC
>Dotsep-cf0203-AA68-REV(ds05015)
GGGCGGAAGGCATCGTCTGTCTTGGCCATGCCCGCGGCCGATCCATTCGCCACAGCAAATACCAAGACTTCCCCC
CGTGGTTCTCGAGTATGGCTAACACCCTTCTGTTTC
>Pasper-cf0203-AA69-REV
GGGTGGAAGGCATCGTCTGGCTCAGACATGTAACCTGCGTCCCCCCCTTTGATAAGGCAGTTGCCGAGACACTGCC
ACATAATGTTCGAGTATGGCTAACACCCTTCTGTTTC
>Pascap-cf0203-AA70-REV
GGGTGGAAGGCATCGTCTGAGACATGGCTTCCCGCCTCCAGCCCAGCAACTGCTGCTGAGACATTGCCACGTGGT
ATTCGAGTATAGCTAACACCCTTCTGTTTC
>Fulful-cf0203-14-1-6-33 (*cf02012* and *cf07001* in Fulfu189536)
GGGCGGAAGGCATCGTCTGTCTGGAACATGCGCGCGGCATTCCCAGCTTACTCGAGGCTGCTGAGACATTGCCAC
GTGGTGTTCGAGTATGGCTAACACCCTTCTGTTTCAGGGATTCGACAAGCTCAGTGCCAATCTCTTTACCCTAGT
ATTAATGGCCAATGCAATCACCTCAGAGCGGAAGGATGCAGATTACCTGTTCCACAAGGGCAGCGCGAAGGAGCG
CTACCAGTTAGTGTCGAACCTCCGTCAGAGTGCCTTTTTCTGGTACGTTACTGAAGGGTACTAGGCATCGGGCAG
TGGCTTAGACCACAATGGAGGGTGCAAGACATGCGCTGCACGACGCGCTCTCTCTACCATTTACACTACAGTCTA
AGACACCGCCACGTGGTTCTCGAGTATGGCTAACACCCTTCTGTTTC
>Pasmiu-cf0203-14-2-2-33-FW
CATCGTCTCTATGAGACATGCGCACAGCACCTCCACTTTATTGGCACTTGCTGAGACACTGCCACGTGGTTCTCG
AGCATGGCTAACACCCTTCTGTTTC
>Pasmiu-cf0203-14-2-2-35-REV
GGGTGGAAGGCATCGTCTGTCTAGAACATGTCCGCACCTCACTCCTCTTCAGCATCACCAGCTGAGACATTGCTA
CGTGGTGTTCGAGTATGGCTAT
>Pasbra-cf0203-14-7-3 (inserted in Dotse59237)
GGCGGAAGGCATCGTCTGTCTGAGACATGCGCGCTGCATCTCCAGCTCAGTAGCCGCTGCCGCGATATTGCTACG
TGGAGTTCGAGTATGGCTAACGCCCTTCTTTCCAGGCTCACCACCAAACTCTCCCTCGGATGCGACGCCACATCC
AGGACTGCCACTCCTGGTCTTGGCCCAGAACTTGGCCTGGATGGTCACAACAAGTTCGAGGGTGATACCTCCCTG
ACACGCAATGACTACTTCACTGCTGGTGGTGTAAGTAAATCCGAAGGGCAGAAGGCATCATCTGTCTCGAGACAC
GCGCGCAGCATATTCAGCTCGATAGCCGCTGCTGAGACATTGCCACGTAAAGTGCAGTATGGCTAACACCCTTCT
GTTTCA
>Fulful-cf04ds03-10-1 (*cf04005*)
CCTAGGGAGTACATACAGTTGTGGCGGAGCTAGCAGTGTCTCAAAACTGCTCACTTGTCTAATACGCACGACAAT
GCGCGCAACAGTAGGCCCCTATAGGGTCGTGCTGAGAGAGA
>Amyafr-cf04ds03-10-6-41-REV
CCTAGGGAGTACATACAGTTGTGGGCCAGGCTAGGCAACGTCTCATAGCGTGGCGTAAGCGTCTAGACAC
