# Supplementary material for: Novel Introner-Like Elements in fungi Are Involved in Parallel Gains of Spliceosomal Introns
Source: PLoS One. 2015 Jun 5;10(6):e0129302. doi: 10.1371/journal.pone.0129302 (PMC4457414; doi:10.1371/journal.pone.0129302)
Supplement: S3 File — (PDF) [file pone.0129302.s003.pdf]

S3 File. DNA and protein alignments built to determine intron landscapes.

Alignments were performed using Muscle as described in the materials and methods section. Exonic sequences from *Passalora* species were aligned to the previous DNA alignment using Muscle and new alignment was manually edited in GeneDoc. All names are as described in Figure 3 and S4 File.

DNA alignments: introns are highlighted in yellow. Four-level shade indicates nucleotide conservation over 90%, 80%, 60%, and under 60%. In D, two-level shade indicates nucleotide conservation over and lower than 65%. In B, insertion of a single nucleotide in the sequence of *Lentfl* makes it a pseudogene.

Protein alignments: positions of RSIs, previously identified ILEs and novel ILEs are indicated in yellow, orange and red, respectively. For novel ILEs, the position is indicated within the sequence of the two most closely related fungal species. Numbers indicate phase 0, 1 or 2. Numbering of intron positions in Figure 3 corresponds to the position in the corresponding alignment below, minus the number of upstream intron positions. Four-level shade indicates amino acid identity of 100%, over 80%, over 60%, and under 60%.

DNA alignment for Figure 3A

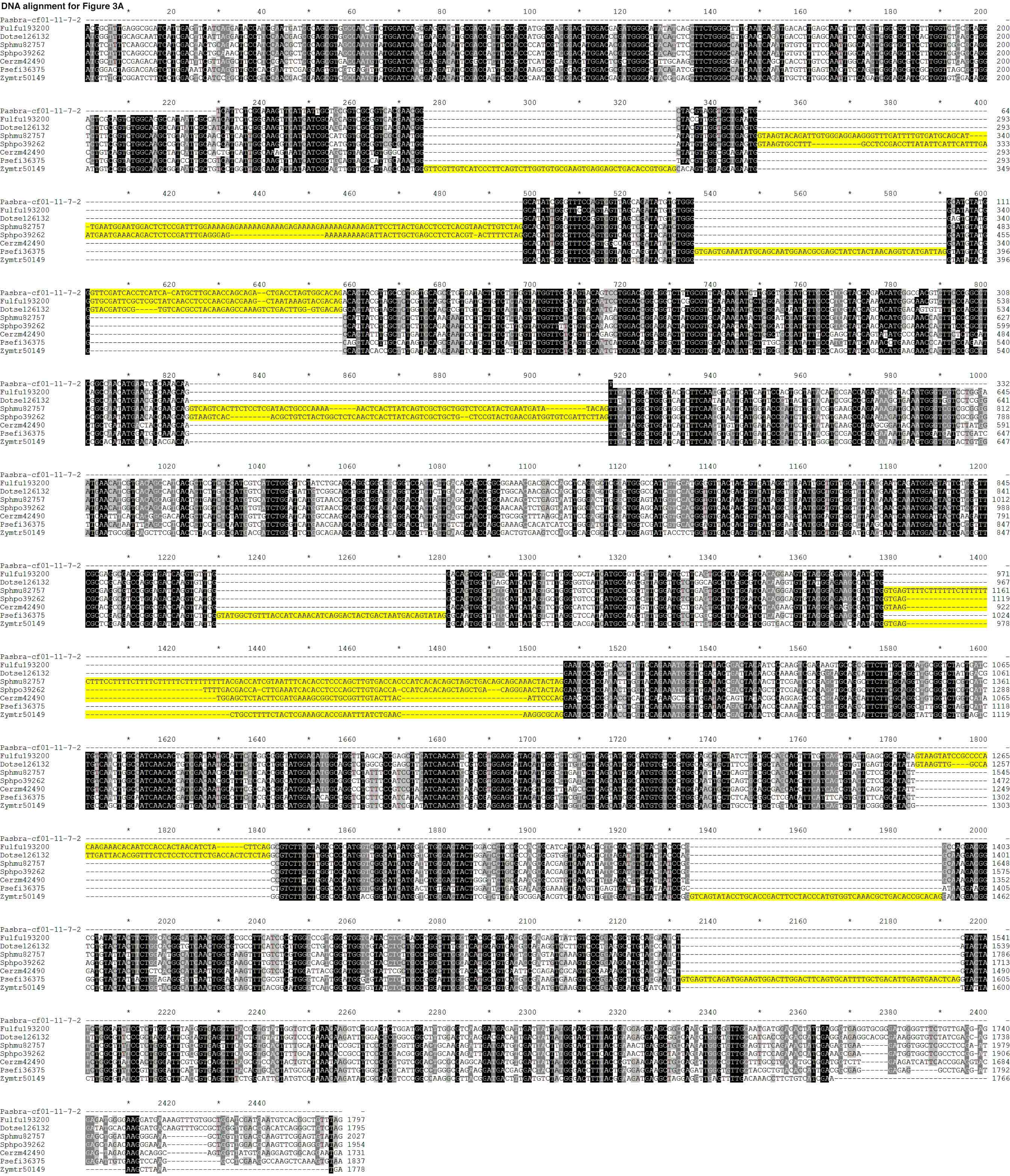

Protein alignment for Figure 3A

```
                      *        20         *        40         *        60         *        80         *       100         *       120         *       140         *       160         *       180         *       200
Fulfu193200  ---------------------------------------------------MTLSNFQLGASLVSAGLAVWQAIIA1IILGKFIIAAVAVTNG-YLGAE-WHIGFPVVSRYVW-GIYG0HYVALVQRVILSLVWFAVQSWTGGLGVQNILAAIFPSYQNMGNVFFASANMNAKQ1FIGWVLFNVLIIPLLYIRPEKLQWV 143
Dotse126132  MGLQQFIDFIRIPSNERSVANVWINEDLRPLPPWRRTWTRWAYISFWALNQMTLSNFQLGASLVSAGLAVWQAIIA1IILGKFIIAAVAVTNG-YVGAE-WHIGFPVVSRYVW-GVYG0HYVALVQRVVLSLVWFAVQSWTGGLCVQNILAAIFPSYQHMDSVFFASANMNAKQ1FIGWVIFNILIVPLLYIRPEKLKWV 194
Sphmu82757   MSFCAIIDYCRLPSNARSVANVWINEDIRPLPPHRRTWTRWAYISFWAINQMCLSNWQLGASLVAAGLSVWQAVIA-TFIGKLIIAMVAVANG-YVGAE2WHIGFPVVSRYIW-GMYG-HYLALFQRILLSLVWFSVQSWTGGLCVQNILASMFPSYQHMGNHFFASANMNTKQ0FIGWVVFNVLMVPILYVKPEKMQWV 194
Sphpo39262   MSFCAIIDYCKLPSNARSVANVWINEDIRPLPPHRRTWTRWAYISFWAINQMCLSNWQLGASLVAAGLSVWQAVIA-TMIGKVIIAMVAVANG-YVGAE2WHIGFPVVSRYIW-GMYG-HYLALFQRILLSLVWFSVQSWTGGLCVQNILASMFPSYQHMGNHFFASANMNTKQ0FIGWVVFNVLMVPILYVKPEKMQWV 194
Cerzm42490   MAFRDILDFVRLPSDSRSVTNVWINEDIRPLPPHRRTWTRWAFASFWAINQLTLSNWQLGASLVSAGLSVWQAIIA-TVIGKVIIAAVAVGNG-YVGAE-WHIGFPVASRYIW-GMYG-HYLALIQRIVLSLVWFSVQSWTGGLCVQNILAAIFPSYQYMPNHFSASADMTTKQ-FIGWIIFNLIMIPILYIKPERIQWV 194
Psefi36375   MGLGRVLEIIRLPTNSRSVVSFWINEDIRPLPPSRRTWTRWAYISFWAINQICLSNFQLGASLVSAGLAVWQAILA-VIIGKFIIAAVAIANG-YVGAE-WHIGFPVVSRYIW1GMYG-SYLAIVQRIILSLVWFSVQSWTGGLCVQNILCAIFPSYQNLKNPFFESAKMLAKQ-FVGWIIFNVLMIPILYVRPEKMKWI 194
Zymtr50149   MFVGSFLESIRLPSNDRSVANVWINEDIRPLPPNRRTWTRWAYVSFWAINQIALGNFQIGASLVSTGLSVWQAVIA-VLVGKIIIAVVAVANG0TVGAE-WHIGFPVVSRYIW-GIYG-HYTALIQRILLSLVWFSVQSWTGGLCVQNILAAIFPSYQHMKNHFFASAHMDTRQ-FIGWIIFNLIMIPILWVRPEKMKWV 194
Glotr82366   --MESGLRFLQVHSSGDE-TSVWINDDIRPLPPSRRTWDKLAFISFWAINQIALSNWQTGSSLVAVGLSVWQTMIA-VIVAKIIISLVAIYNG-YVGAE-WHIGFPVVSRYVW-GVYG-SYLSLLQRILLSLVWFSVQSWTGGLCITAIISSIFPSFQHMGNTMFASTHMTTKQ-FVGWVVYNVVTVPMLYIPPEKTKRL 191

                      *       220         *       240         *       260         *       280         *       300         *       320         *       340         *       360         *       380         *       400
Fulfu193200  VLGMNIVTAITLLSIVIWVLSAAGGGGPLLTQFAETTTSSELGWAIVHGVTTVIGGIAVGLTNQMDYSRFARRPGDQVF-GQWFSIIVFGAIMPSLGCFSASASQEVYGEAI-WNPPDLVQKWLDTDYNPKSRSAAFFAGCGLLICQLAINTVDNAFSAGMDMAGLAPSFINIRRGAYIGLVLSIAMCPWQLLSAASTFV 341
Dotse126132  VLGMNIVTAITLLSMVIWVLSAAGGGGPLLTQFAGTTTSSELGWAIVHGVTTVIGGIAVGLTNQMDYSRFARRPGDQVF-GQWFSIIVFGVIMPALGCIAASASQGVYGEAI-WNPPNLVQKWLDTDYNAKSRAAAFFAGCGLLTCQLAINTVDNAFSAGMDMAGLAPSFINIRRGAYIGLVLSIAMCPWQLLSAASTFI 392
Sphmu82757   VAGMNMVTAVTLISMCIWIMVTAGGGGPLLSQFATALSNWDLGWSMVHGVTTVIGGIAVGLTNQMDYSRFARRPGDQVM-GQWLSIIVLGVLMPALGCLSASASQGVYGEAI2WNPPNLVQKWLDTDYNSRSRAAGFFAGCGLVICQLAINTIDNAFSTGMDMAGLFPSFINIRRGAYIGLILSIAMCPWQLLSAASTFI 392
Sphpo39262   VAGMNMVTAVTLVSMCIWIMVTAGGGGPLLSQFATTLSNWDLGWSMVHGVTTVIGGIAVGLTNQMDYSRFARRPGDQVM-GQWLSIIVLGVIMPALGCLSASASQGVYGEAI2WNPPNLVQKWLDTDYSSRSRAAGFFAGCGLVICQLAINTIDNAFSTGMDMAGLFPSFINIRRGAYIGLILSIAMCPWQLLSAASTFI 392
Cerzm42490   VLWFNFITATTLISMVVWIMAKAGGGGPLLSQFAAALSNSELGWLMVHGVTTVIGGIAVGLTNQMDYSRFARRPGDQVM-GQWLSIIVLGIFMPSLGCLSASASQKVYGEAI2WNPPNLIQKLLDTSYTPGARAAAFFAGCGLVICQLAINTIDNAFATGMDMAGLFPSYINIRRGAYVGLALSIAMCPWKLLSAASTFI 392
Psefi36375   ILIFNIISALTLLSMVIWVMNEAGGGGPLLSQFAKATSSWDLGWSIVHGVTTVIGSIAVGLSNQMDYSRFARRPGDQVF1GQWFSIIVLGSIMPALGCLGASASQAVYGEAI-WNPPNLVQKWLDTDYNPKSRAAAFFAGFGLVICQLAINTIDNAFSAGMDTAGLFPSYINIRRGAYIGLVLSIAMCPWELLSAASTFI 392
Zymtr50149   VLWMNAVSFVTLLAMTIWLLAEAGGAGPLLKQFATVKSSSALAWSITSGVTTVIGGIAVGLTNQMDYSRFARRPGDQVI-GQWVSIIAFGTIMPTLGCLCASASVTVYGEPI2WNPPNLVQKWLDTDYTAKSRAAAFFAGIGLVVCQLAINTIDNAFSTGMDMAGLFPSYINIRRGAYVGLVLSIAMCPWELLASAGTFI 392
Glotr82366   FIVMNVISFFTLISMMIWALSQAHGGGPLLSQFAAVSSSSELGWAIVSGITTVIGSIAVGLTNQPDYSRFARKPGDQIF-GQWFSIIFFGTIMPLFGCLTSSASVAIYGEAL-WNPPDIVLKWLDTDYTAKSRAAAFFAGVGLVVCQLAINTIDNAFSAGMDIAGLFPKYINIRRGAYIGLVLSIIMQPWQLLSSAGTFI 389

                      *       420         *       440         *       460         *       480         *       500         *       520         *       540         *       560         *
Fulfu193200  SVLSAY1SVFLGPMVGIMVCDYWTLRHRIIKLSDLYHP-GQDGLYYFWHGINWRAFIAWAVGWSYLLPGFAHAVTPRVIVPEACTN-LYYLAFPLGFTVSFTVYWCLNKVWTLDGFGVKDEIDYYGTFTEEEAAKLGVAMMETIEGEVRDGVSVEEEMGKDEKFVAGSMNVTAV 512
Dotse126132  SVLSAY1SVFLGPMVGIMVCDYWILRHRVVKLSDLYHP-GKDGLYYFWHGVNWRAFIAWAVGWSYLLPGFAHAVTPKVLVPEACTH-LYYLAFPLGFAVSFTVYWSLNKIWTFAGFGVKDDIDYYGTFTEEEAAKLGVAMTETIEGEARERVSIEQDMHKDDKFAAGLTDIRAV 563
Sphmu82757   SVLSAY-SVFLGPMVGIMICDYFILRKRRVKLSDLYHP-GKDGLYYFWNGINWRSFVSWAIGWSYLLPGFAHAVTPSIKVPEACTN-LYYLAFPLGFAVSFSVHFCINTAFPPVGLQEIDDVDYFGTFTETEAAKMGVAVSETIESEDGGSPPNELDKGK-----AGLTQVRSV 558
Sphpo39262   SVLSAY-SVFLGPMVGIMICDYFILRKRRVKLSDLYHP-GKDGVYYFWNGINWRSFVSWAVGWSYLLPGFAHAVTPIVKVPEACTN-LYYLAFPLGFAVSFSVHFCINTAFPPRGLQEIDDVDYFGTFTETEATRMGVAVPETIESEDGGSPPDELDKGK-----AGWTQVRRV 558
Cerzm42490   SVLSAY-SVFLGPMVGIMICDYWVLRKRRVKLSDLYHP-GKDGIYYFSHGINWRSFVAWITGWSYSLPGFVHAVNSEIAVPKACTN-LYYLAYPLGFVVSFSVHYCLNKAFPPVGLREADDVDYYGTFTGEEAARLGVALPEMLDGLEDHSPEVRVEKTG-----SGYVKEWQE 558
Psefi36375   SVLSAY-SVFLGPMVGIMTCDYWILRKRKVKLSDLYNP-HKEGVYYYWRGINWRSFASWVIGWAYLLPGFAHAVTPKVKVPEACTN2LYYLAFPLGFTVSFTVHYAINKLWPPIGLGEKDDEDYYGTFTEEEAARLGVATLYTIDGEER--PEDEIVKSK-----ASKAKLKV- 555
Zymtr50149   SVLGAY-GVFLGPMTGIMVCDYFVLRRRRLKLSDLYHP1GKDGLYYFWYGINWRSFTAWVIGWCYLLPGFAHAVTPNVKVPEAWNH-LYYLAYPLGFTVSFLAFYVLNKISPPPGQGVTDDFLVYGTFTEDEARRLSFDKPSVIEKLK-------------------------- 537
Glotr82366   SVLSAY-SVFLGPMVGMQVCDYWLVRGRKIKLSDLYSS-SPEGIYYSRGWNWRAFAAWIVGWASQIPGFVNAVNPDIKVPVGSQH-LYYLAFPAGFCISFLVFYALNRLAPPAGLGEVDDVDYFGTFDEEEADKMGIRPAFDSDSSGADVEVDEKRLDL-----ERTVSVDKL 555
```

DNA alignment for Figure 3B

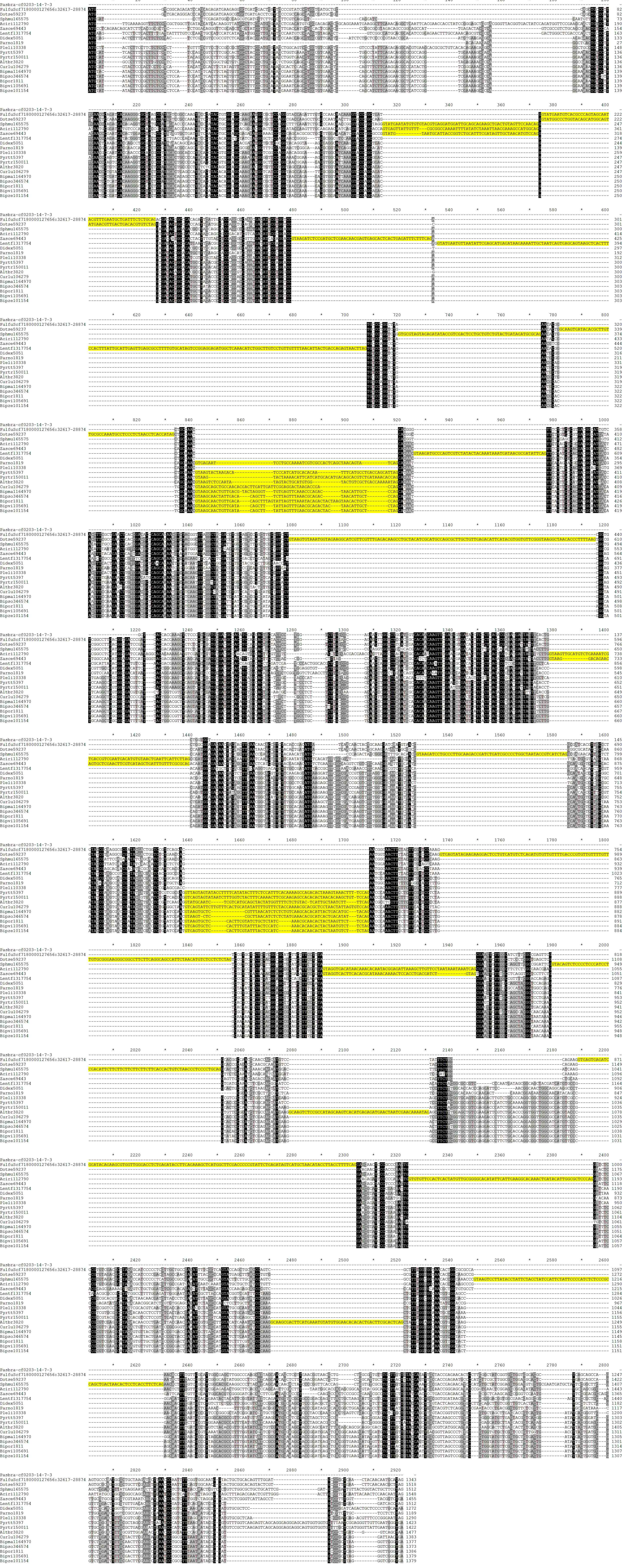

Protein alignment for Figure 3B

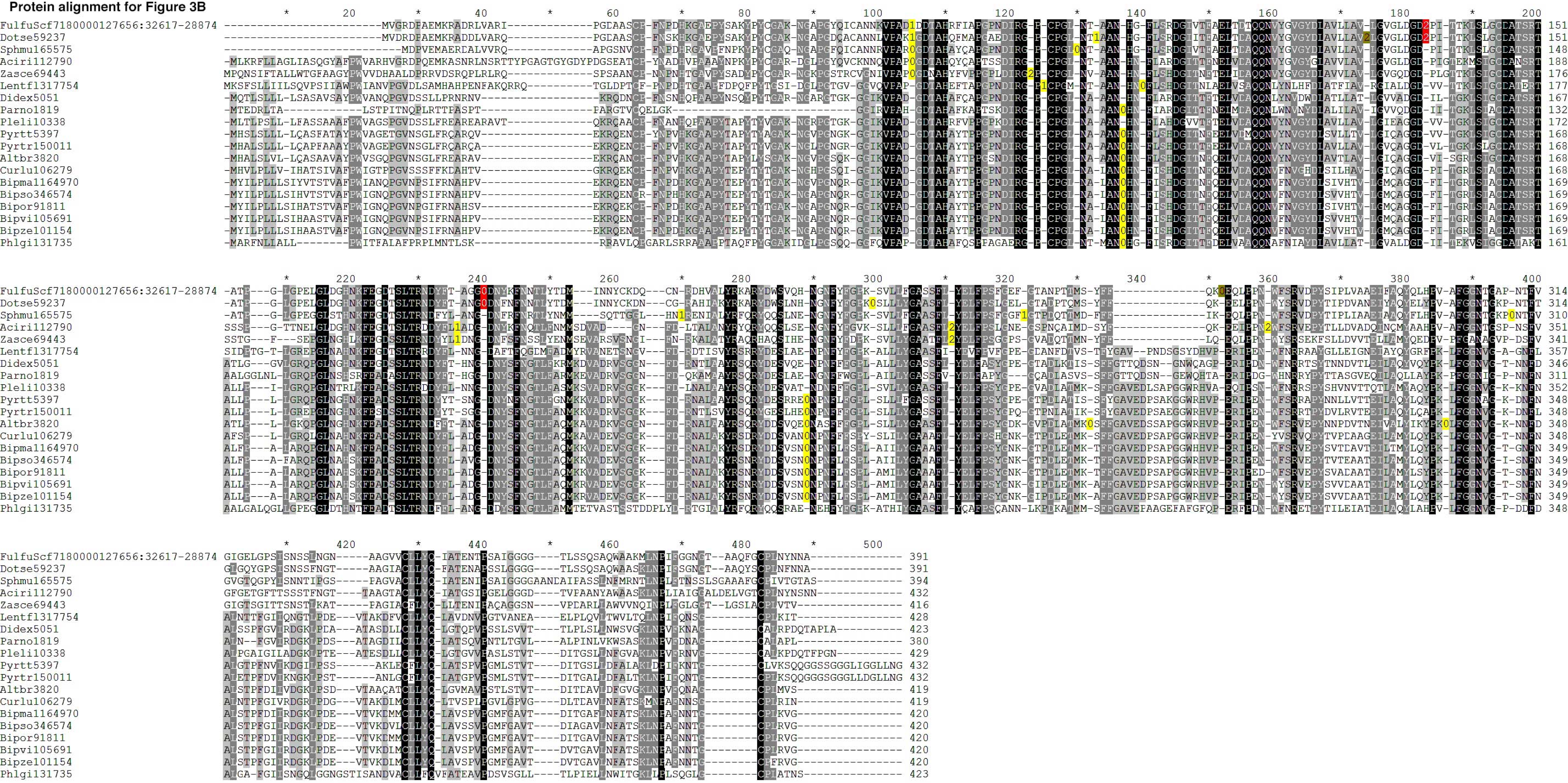

DNA alignment for Figure 3C

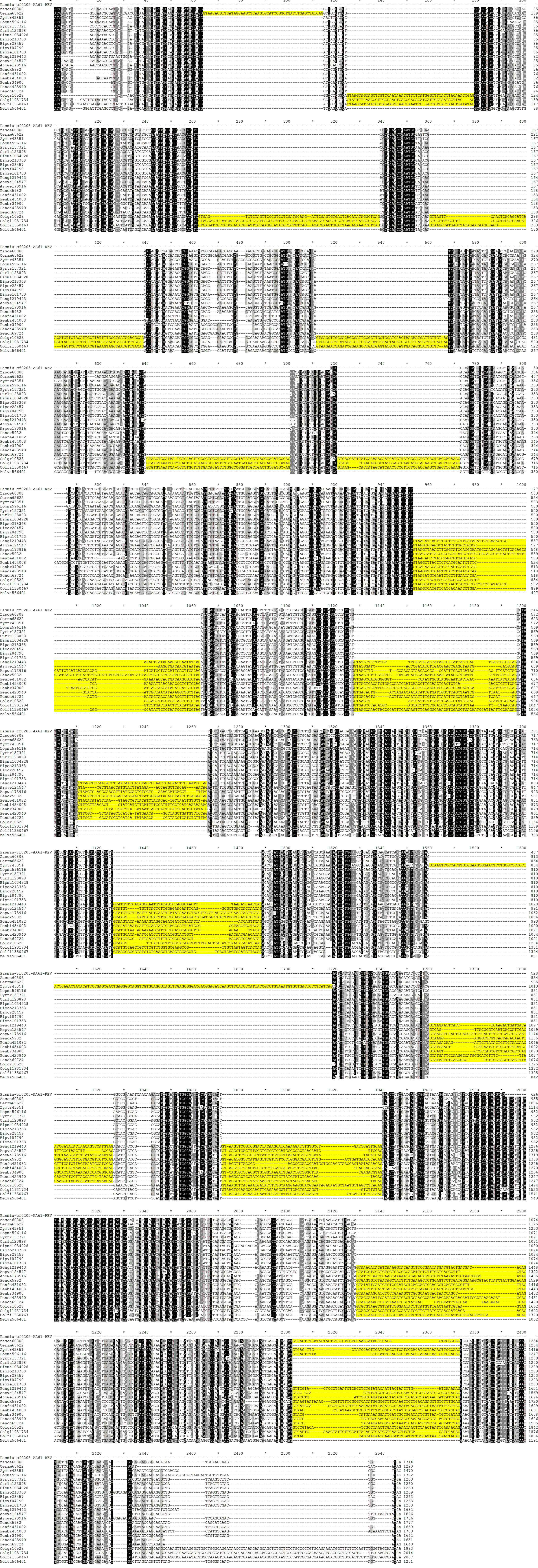

Protein alignment for Figure 3C

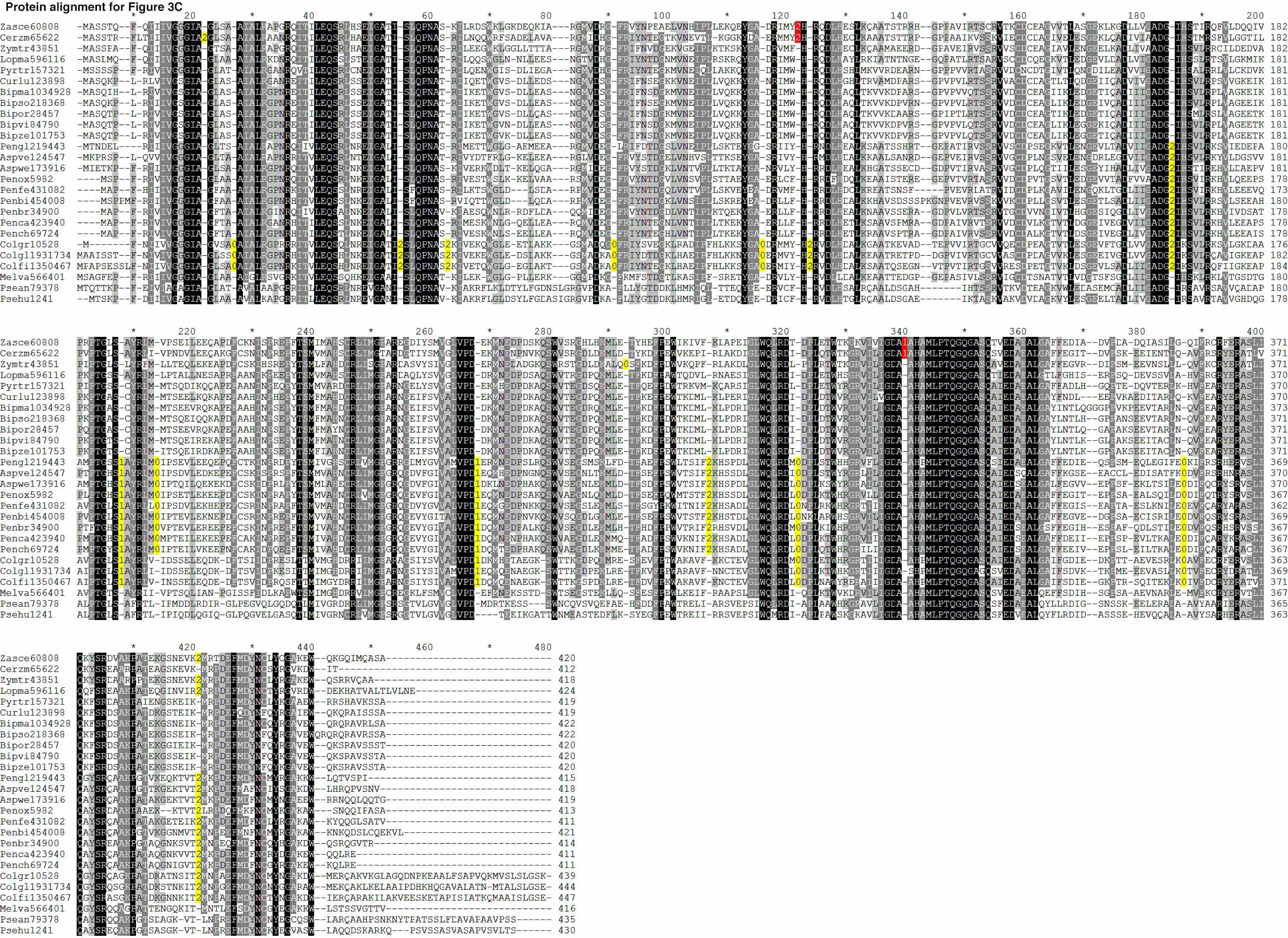

DNA alignment for Figure 3D

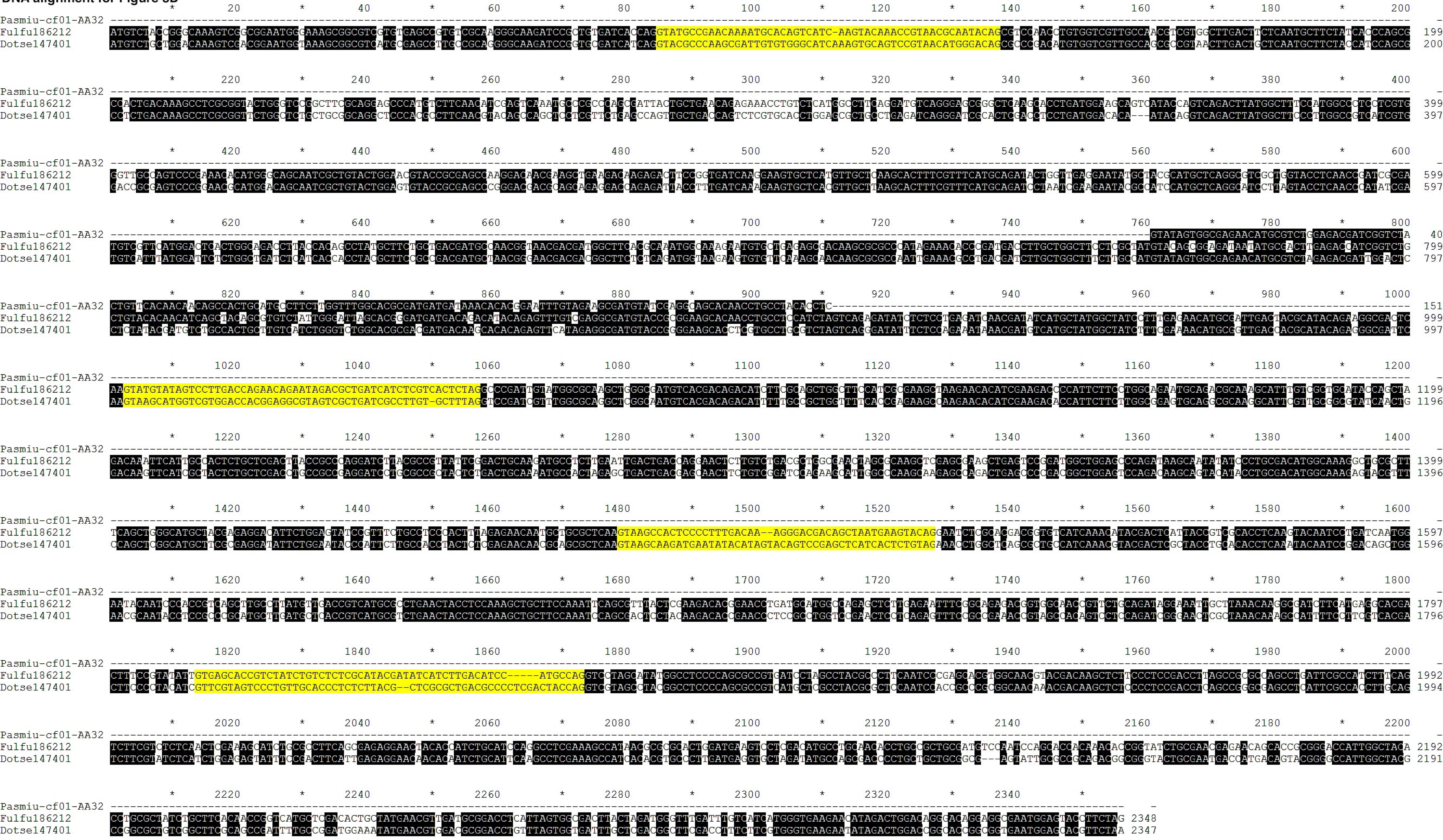

Protein alignment for Figure 3D

```
                        *        20         *        40         *        60         *        80         *       100         *       120         *       140         *       160         *       180         *       200
Fulfu186212 MSTGQSRRNGKAASCEPCRKGKIRCDHQ0RPTCGRCQRRGLTSQCFYHFAPLTKPRGTGSGFAGAHVFNIESNARPAITAEQRNLSHGLQDVRERAQAPDGSSHTSQTYGFPWPSSWVASPETHGQQSLYWNVPRAKDNEAEDKRLPVIKEVIMLLKHFRFMQILVEEYATHAQASLVPQPIAMSFMDSLADLTTAYASA 199
Dotse147401 MSAGQSRRNGKAASCEPCRRGKIRCDHQ0RPTCGRCQRRNLTAQCFYHFAPLTKPRGSGSAAAGSHAFNVQPAPRSEPVADQSRAPGALPEIRDRTRPPDGHN-TGQTYGFPWPSSWTASPGTHGQQSLYWSVPRARDDAAEDQRLPLIKEVLTLLKHFRFMQILIEEYAIHAQASLVPQPISMSFMDSLADLITTYASA 198

                        *       220         *       240         *       260         *       280         *       300         *       320         *       340         *       360         *       380         *       400
Fulfu186212 DDANGNDDGFTQMAKNVLRATSAPIETPDDLAGFIA2MYSGDNMRLETIGLLYTTSATACLLGLARDDDRHTEFVEAMYRGSTTCLH2LVRDISPEINDIMLWLSFENMRLTTHTEGDS1SPIVWRKLGDVTTDIFAAGFHREAKNTSKTPFFLAECRRKAFVAAYQLDKFIATLLDLPPRILRRYSDCKMPLELTDQEL 396
Dotse147401 DDANGNDDGFSQMVRSVFKATSAPIETPDDLAGFIA2MYSGENMRLETIGLLYTMSATACHLGLARDDDKHTEFIEAMYRGSTSCLR2LVRDISPEINDVMLWLSFENMRLTTHTEGDS1SPIVWRRLGNVTTDIFAAGFHREAKNTSKTPFFLAECRRKAFVAAYQLDKFIATLLDLPPRILRRYSDCKMPLELTDEQL 395

                        *       420         *       440         *       460         *       480         *       500         *       520         *       540         *       560         *       580         *       600
Fulfu186212 LSDAGELAQARAKLSPDGWSPDKQYIFATWQRLRFQLGMLREDILEYPFLPPTLENNAAL2KNLARRCHQTYDSLPSHLKYNPDQWNTIPPSACLMLTVMRLNYLQSCFQIQRLLEDTEPDAWPELLRISAETVATVLQIGNCLNKAIFMRHDFPYI0VLAYGLPSAVILAYALQSRARGNVRCALPSDLSRASLIRHLS 594
Dotse147401 LSDPEALAQARARLSPDGWSPDKQYIFATWQRVRFQLGMLREDILEYPFLPPTLENNAAL2KNLAQRCHQTYDSLPAHLKYNPDSWNAIPPPACLMLTVMRLNYLQSCFQIQRLLQDTEPSAWSELLRVSAETVATVLQIGNSLNKAIFLRHDFPYI0VVAYGLPSAVMLAYALQSTARGNKRCALPSDLSRASLIRHLA 593

                        *       620         *       640         *       660         *       680         *       700         *       720
Fulfu186212 VFVSQLESICAFSERNYTICIQASKAITRALDEVLDMPATFAAAMSNPAPQTPVSANENSTAGPLATPALSASQPVMLDTAMNVDADLISGDLLDGFDLSSWVKNIDWTGTGGEWSTF-- 712
Dotse147401 VFVSHLESISDFIERNNTICIQASKAITRALDEVLDMPATFAAA-ASIAPQTAGTANDHDSTGPLATPALSASQPILPDGNMNVDADLFSGDLLDGFDLSSWVKNIDWTGTGGEWSTF-- 710
```
