# Supplementary material for: Novel Introner-Like Elements in fungi Are Involved in Parallel Gains of Spliceosomal Introns
Source: PLoS One. 2015 Jun 5;10(6):e0129302. doi: 10.1371/journal.pone.0129302 (PMC4457414; doi:10.1371/journal.pone.0129302)
Supplement: S1 Table — (PDF) [file pone.0129302.s004.pdf]

S1 Table. GenBank accession numbers of the ITS and LSU sequences used in this study.

Strain accession numbers correspond to the CBS-KNAW collection (CBS) or culture collection of Pedro Crous (CPC).

Fungal species	Strain accession number	ITS sequence accession number	LSU sequence accession number
Amycosphaerella africana	CBS 116154	AF173314.1	DQ246257.1
Passalora brachycarpa	CBS 115124	GU214664.1	GQ852619.1
Passalora microsora	CBS 123735	KJ633262	KJ633266
Passalora perfoliati	CBS 113378	DQ676520.1	DQ676520.1
Passalora daleae	CBS 113031	EU040236.1	EU040236.1
Passalora capsicicola	CBS 156.62	KJ633263	KJ633267
Passalora miurae	CPC 14643	KJ633264	KJ633268
Passalora smilacis	CBS 556.71	KJ633265	KJ633269
