# Supplementary material for: Novel Introner-Like Elements in fungi Are Involved in Parallel Gains of Spliceosomal Introns
Source: PLoS One. 2015 Jun 5;10(6):e0129302. doi: 10.1371/journal.pone.0129302 (PMC4457414; doi:10.1371/journal.pone.0129302)
Supplement: S2 Table — (PDF) [file pone.0129302.s005.pdf]

S2 Table. Primers used in this study.

Name	Sequence (5' to 3')
Cf01-F	CTGAAGGGTGCTAGGCATCTTCG
Cf01-R	AAAAGCAAAGGGTACAAGGT
Cf02-F	CAGAAGGCATCATCTGTCTGAAA
Cf02-R	CAATGTCTCAGCAACTGCTACTGA
Cf0203Ds0105-F	GGGYRGAAGGCATCRTYT
Cf0203Ds0105-R	GAAACAGAAGGGTGTTAGC
Cf04Ds03-F	CCTAGGGAGTACATACAGTTGTG
Cf04Ds03-R	TCTCTCTCAGSACRMCCCTATAGG
Cf08Ds04-F	TTGTAGRGTRTACCTAWCGCATCGT
Cf08Ds04-R	ACCAGCACGTCAYCRTGAGA
